# Metaheuristic-Driven Machine Learning for Best-Observed Feasible Weight Estimation of Braced Steel High-Rise Frames

**DOI:** 10.3390/ma19204272

**Published:** 2026-10-09

**Authors:** Betül Üstüner, Aybike Özyüksel Çiftçioğlu, Erkan Doğan, Ali Mardani

**Affiliations:** 1 Department of Civil Engineering, Faculty of Engineering and Natural Sciences, Manisa Celal Bayar University, Manisa 45030, Türkiye; betul.celik@cbu.edu.tr (B.Ü.); aybike.ozyuksel@cbu.edu.tr (A.Ö.Ç.); erkan.dogan@cbu.edu.tr (E.D.); 2 Department of Civil Engineering, Faculty of Engineering, Bursa Uludag University, Bursa 16059, Türkiye

**Keywords:** high-rise steel structures, bracing systems, structural optimization, metaheuristic algorithms, machine learning, weight prediction

## Abstract

Preliminary selection of bracing systems in high-rise steel buildings requires comparing many configurations, each demanding computationally intensive minimum-weight design. This study uses machine learning to estimate the best-observed feasible weight directly from building-level configuration parameters. A full factorial dataset of 45 configurations covered three storey numbers (17, 24, and 30), three bracing types (V, X, and K), and five brace-placement layouts. Each configuration was optimized in a coupled SAP2000–MATLAB procedure with Particle Swarm, Grey Wolf, Honey Badger, and Aquila optimization (180 runs in total); no single algorithm was best for every configuration. Mean weight increased from 2537 kN at 17 storeys to 7103 kN at 30 storeys. X-bracing gave the highest mean weight at all heights and K-bracing the lowest at 17 and 24 storeys, whereas V- and K-bracing differed by only 2.4% at 30 storeys. Bracing type changed the mean weight by up to about 30%, and brace placement by about 10–15%. Among six regression models, Ridge achieved the highest mean five-fold cross-validated *R*^2^ (0.914; 0.904 over 1000 repeated partitions), while Random Forest gave the lowest RMSE (511.7 kN); in leave-one-out prediction, Ridge ordered 90.3% of configuration pairs correctly. The resulting Ridge equation offers an interpretable screening tool within the investigated design domain.

## 1. Introduction

As building height increases, lateral actions become increasingly important in the design of steel framing systems. Wind and earthquake effects increase storey drift, overturning demand, and the axial and flexural forces carried by structural members. The distribution of lateral stiffness over the building height therefore becomes a major consideration in the design of tall buildings. Steel is widely used for such structures because its high strength-to-weight ratio, prefabrication potential, and construction efficiency permit relatively slender structural systems without an excessive increase in self-weight [1]. Different lateral load-resisting systems have consequently been developed, including braced frames, shear-core systems, and outrigger arrangements [2,3]. Among these alternatives, concentrically braced frames provide a direct load path in which a substantial portion of the lateral demand is resisted through axial forces in the diagonal members. Their apparent geometric simplicity, however, does not result in a simple design problem. Brace type, vertical brace distribution, member sizing, and building height jointly affect lateral stiffness, drift demand, and the redistribution of forces among structural members. Consequently, a configuration providing greater lateral stiffness does not necessarily lead to the lowest structural weight once strength and serviceability requirements are imposed [4].

The vertical distribution of stiffness is particularly important in tall buildings. In outrigger systems, the location of the outriggers governs the extent to which overturning moment is transferred from the central core to the exterior columns and therefore directly influences lateral displacement and internal-force distribution [5,6]. Previous studies addressing the number and location of outriggers have shown that the most favourable arrangement is not universal but changes with building geometry, stiffness characteristics, structural system, and design objective [7,8,9,10]. A similar behaviour is expected in braced frames. Changing the storeys at which braces are installed alters the vertical stiffness profile of the frame and, in turn, its deformation pattern, inter-storey drift distribution, and the forces transferred among braces, beams, and columns. These changes affect the member sections required to satisfy the design constraints and ultimately influence the total structural weight. Brace placement should therefore be considered together with member sizing rather than being fixed independently before optimization.

Minimum-weight design of steel frames is complicated further by the discrete nature of commercially available steel sections and by the simultaneous enforcement of strength, displacement, drift, and member-compatibility requirements. Structural optimization has consequently progressed from simplified analytical and gradient-based procedures towards finite-element-based searches and population-based metaheuristic algorithms [11,12,13,14,15]. Metaheuristic algorithms are particularly suitable for section-selection problems because they can search discrete design spaces without requiring derivatives of the objective function. Brace location and topology have also been addressed within this class of optimization problems, and previous studies have demonstrated that changes in brace arrangement can alter both structural response and the resulting optimum design [16,17]. Experimental and numerical investigations similarly indicate that brace configuration affects global stiffness, force transfer, and member demand [18,19,20]. The preferred brace arrangement may therefore change as the height and stiffness distribution of the structure change, making both bracing type and brace placement relevant variables in minimum-weight design.

The main practical difficulty is the computational effort required to compare these alternatives. During metaheuristic optimization, each candidate combination of member sections must be assigned to the structural model, analysed under the prescribed load combinations, checked against the design constraints, and then retained or penalized according to its performance. This process is repeated for many candidate designs over successive iterations. The computational burden becomes more pronounced at the preliminary design stage, where several structural alternatives must be compared rather than a single predetermined configuration optimized. Previous minimum-weight studies on real-scale high-rise steel buildings have shown that repeated evaluation of alternative stiffening arrangements can require a substantial number of structural analyses [21]. In the present study, the combination of three building heights, three bracing types, and five brace-placement layouts gives 45 structural configurations. Applying four metaheuristic algorithms to every configuration requires 180 optimization runs, each using a population of 30 candidate solutions and a maximum of 50,000 iterations. The motivation for the present study arises directly from this computational demand. If the relationship between readily defined configuration parameters and the best-observed feasible weight can be learned from completed optimization runs, promising alternatives may be screened before undertaking further optimization and detailed structural design.

Machine learning (ML) provides a means of extracting such relationships from previously analysed cases. During the past decade, ML methods have been used in structural engineering for capacity prediction, failure assessment, fire-performance evaluation, and design-related prediction problems [22,23,24,25]. Bekdaş et al. [26] trained ML models using the results of metaheuristic optimization to estimate optimum design variables, although the applications considered comparatively simple structural systems. Shan et al. [27] later combined optimization and ML within an intelligent design procedure for steel frames, while the optimization stage remained necessary when a new design problem was considered. Truong et al. [28] used an XGBoost surrogate to approximate computationally demanding constraint evaluations in bi-objective optimization of nonlinear steel structures. This reduced the number of expensive structural analyses, but the surrogate remained within the optimization loop. ML-based approaches have also been combined with genetic algorithms for the design of high-rise outrigger systems, mainly with emphasis on seismic performance [29], and regression and classification surrogates have been investigated for the sizing optimization of nonlinear steel trusses [30]. A recent review of ML applications to steel–concrete composite structures further shows that regression and tree-based ensemble methods are increasingly being used to estimate structural response and design-related quantities from parametric datasets [31].

The ability of prediction models to reduce repeated calculations has also been demonstrated in other civil-engineering problems. Mardani-Aghabaglou et al. [32] used artificial intelligence and regression approaches to predict cement-paste flow behaviour from material and mixture parameters, while the influence of cement characteristics on rheological response has also been examined using regression-based modelling [33]. Soft-computing models have been applied to the prediction of strength and penetrability characteristics of cement-stabilized materials [34], and different regression algorithms have been compared for estimating thixotropic behaviour from material composition and testing parameters [35]. These studies show that useful engineering relationships can be extracted from datasets containing several interacting input variables, provided that model performance is evaluated against observations not used for fitting.

More recent studies have combined predictive modelling with optimization algorithms. Experimental datasets have been evaluated using regression, multivariate adaptive regression splines, and optimization-assisted artificial neural networks [36]. ANN models trained using biogeography-based optimization have also been employed to estimate the mechanical properties of high-volume fly-ash roller-compacted concrete [37]. In another application, gradient boosting, Ridge regression, and support-vector regression were compared for estimating energy consumption and fineness from clinker-grinding parameters, with model performance assessed by cross-validation [38]. An ANN coupled with biogeography-based optimization was subsequently used to predict both compressive strength and flow characteristics of fly-ash cementitious systems [39], while optimized neural networks and metaheuristic algorithms have been applied to prediction of temperature-induced degradation in structural concrete [40]. Statistical optimization has likewise been used to identify the relative influence of design variables on the performance of fibre-reinforced roller-compacted concrete [41], and ANN, Random Forest, XGBoost, and related methods have been employed to model grinding performance from processing and material parameters [42]. Although these applications concern different engineering responses, they demonstrate a common advantage of prediction models: once a relationship has been established over a defined range of input variables, repeated experimental or numerical evaluations can be reduced considerably.

The literature discussed above indicates that ML has entered structural optimization mainly in two forms. In the first, the learned model replaces an expensive structural analysis or constraint evaluation while an optimization algorithm continues to search for the design. In the second, ML is used to estimate response quantities or design variables from previously generated data. A more direct question remains relevant at the preliminary design stage of braced high-rise frames: whether the outcome of a completed constrained optimization can itself be estimated from a small number of building-level configuration variables. Such a prediction would not merely reduce the cost of an individual structural analysis within an optimization run; it would allow different combinations of building height, bracing type, and brace placement to be compared without repeating the entire optimization procedure for every preliminary alternative.

Three features distinguish the present formulation from the studies summarized above. First, the quantity being learned is the converged objective-function value of a completed constrained optimization, rather than a structural response, a constraint value, or a set of member sections generated during the search. Second, the predictors are building-level descriptors, namely storey number, bracing type, and brace-placement layout, which are fixed at the concept stage, before member sections, internal forces, or a detailed analysis model exist. Surrogate models formulated at member level require inputs that are not yet available at this point in the design process. Third, because the predictors describe the topology of the bracing system rather than continuous sizing variables, the relationship is fitted across structural alternatives rather than within a single configuration, and it is expressed as an additive equation that the designer can evaluate directly. The model therefore addresses the screening stage that precedes optimization, and narrows the set of configurations for which the full optimization procedure is subsequently carried out.

Table 1 summarizes representative studies most closely related to the development from direct optimization of stiffening systems towards ML-assisted structural design. The comparison shows that previous studies have addressed brace arrangement, outrigger location, surrogate evaluation, or ML-assisted optimization, whereas direct prediction of the best-observed feasible weight from high-level bracing configuration parameters has received considerably less attention.

The present study addresses this problem by estimating the best-observed feasible weight of braced high-rise steel frames directly from building height, bracing type, and brace-placement layout. Three storey numbers (17, 24, and 30), three bracing types (V, X, and K), and five brace-placement layouts were combined to obtain 45 structural configurations. Structural analyses were performed in SAP2000 (version 23; Computers and Structures, Inc., Walnut Creek, CA, USA), whereas the optimization was carried out in MATLAB (R2019b; MathWorks, Natick, MA, USA) and the ML calculations in Python (version 3.13.16; Python Software Foundation, Wilmington, DE, USA). SAP2000 and MATLAB were linked through the Open Application Programming Interface (OAPI), allowing section assignments, structural analyses, constraint checks, and optimization results to be transferred automatically. Each configuration was analysed using Particle Swarm Optimization, Grey Wolf Optimization, Honey Badger Optimization, and Aquila Optimization. The lowest feasible weight obtained from these four searches was retained as the target value for that configuration, giving 180 optimization runs in total. The resulting 45 best-observed feasible weights were then used to train and evaluate six regression models.

The study therefore examines three related questions. First, it determines how building height, bracing type, and brace-placement layout affect the best-observed feasible weight obtained from the optimization analyses. Second, it evaluates whether models trained on previously optimized configurations can reproduce these weights with sufficient accuracy and consistency to distinguish between preliminary design alternatives. Third, it examines whether the resulting prediction can reduce the need for repeated optimization when alternative bracing configurations are screened within the investigated range. The resulting model is intended for preliminary comparison of configurations; code-compliant structural analysis and detailed optimization remain necessary for final design. The loading considered comprises gravity and wind actions; seismic design conditions lie outside the scope of the present dataset.

## 2. Materials and Methods

Structural analyses were performed using SAP2000 version 23, while the optimization procedures were implemented in MATLAB R2019b. The machine-learning procedures were implemented in Python 3.13.16 with the scikit-learn 1.6.1 library in the Google Colaboratory environment (Google LLC, Mountain View, CA, USA). SAP2000 and MATLAB were linked through the Open Application Programming Interface (OAPI), enabling MATLAB to assign candidate member sections, initiate the structural analyses in SAP2000, retrieve the required response quantities, evaluate the design constraints, and return the resulting objective-function values to the optimization routine without manual intervention. Beams, columns, and bracing members were modelled as structural steel members using the material properties assigned in the corresponding SAP2000 models, and the column bases were assumed to be fully fixed. The same modelling assumptions were retained throughout all optimization runs. All beams and columns were modelled using ASTM A992 Grade 50 structural steel, while ASTM A500 Grade C steel was adopted for the bracing members. A nominal yield strength *F_y_* of 345 MPa and Young’s modulus *E* of 200 GPa were used for all steel members. Poisson’s ratio and steel density were taken as 0.30 and 7850 kg/m^3^, respectively, corresponding to a unit weight of approximately 77.0 kN/m^3^. These material properties were maintained for all structural configurations and optimization runs. Structural response was evaluated under the prescribed loading conditions described in Section 2.1.2. Wind loading was included in the 24- and 30-storey models but was not applied to the 17-storey models. Second-order effects were included in the structural analysis, and stability was treated by the direct analysis method described in Section 2.1.3. The best-observed feasible weights obtained in this study should be interpreted within the modelling assumptions, loading conditions, and structural configurations considered herein. Beam-to-column connections were modelled as rigid. Moment releases about both local axes were assigned at both ends of every bracing member, so that the braces act as axial members, while the remaining member degrees of freedom were left continuous. No floor diaphragm was defined; the floor loads were applied directly to the supporting beams, and lateral resistance is provided by the moment-resisting frames acting together with the bracing.

### 2.1. Parametric Optimization of Braced Steel Frames

Bracing is widely used to improve the lateral resistance of tall steel frames because diagonal members transfer a substantial part of the lateral demand through axial action, thereby increasing global stiffness without requiring a comparable increase in structural weight [43]. The effectiveness of a braced frame, however, depends not only on the presence of diagonal members but also on their configuration and distribution over the building height [44,45]. Changes in brace arrangement modify the lateral load path, storey-stiffness distribution, internal-force transfer, and deformation pattern of the frame [46,47,48]. These effects become increasingly important as building height increases because the relative contribution of overturning, lateral displacement, and storey-drift demand changes along the height. Previous studies have consequently reported that no single brace arrangement provides the most favourable response for every structural configuration [46,47,48,49]. On this basis, building height, bracing type, and brace-placement layout were treated as the principal configuration variables in the present study, while the cross-sections of beams, columns, and braces were determined through minimum-weight optimization.

#### 2.1.1. Structural Configurations

The structural models consisted of braced steel frames with 17, 24, and 30 storeys. The floor-to-floor height is 3.0 m in the 17- and 24-storey models and 3.66 m in the 30-storey model, giving total structural heights of 51, 72 and 109.8 m, respectively. The centre-to-centre bay spacing equals the storey height in each model. These geometric dimensions were maintained for all bracing types and brace-placement layouts belonging to the same height group. The three height levels were selected to examine the variation in the best-observed feasible weight and in the relative performance of alternative bracing arrangements as the number of storeys increased. For each height, three brace configurations, namely V-, X-, and K-bracing, were investigated as alternative lateral load-resisting arrangements.

The three models are irregular and their plans differ from one another. Each plan was adopted from a frame previously reported in the literature [50,51,52], and only the storey number and the loading were redefined for the present study. The 17-storey model has a cruciform plan on a six-by-six bay grid, giving an 18 m by 18 m envelope; its lower eight storeys contain 33 columns, comprising 12 corner, 12 edge and 9 interior columns, and above the eighth storey the projecting wings terminate so that a central two-by-two-bay block containing nine columns continues to the seventeenth storey. The 24-storey model has a cruciform plan on a nine-by-nine bay grid, giving a 27 m by 27 m envelope, with setbacks at several elevations; its highest projecting portion terminates at the fifteenth storey, above which only the central block continues. The 30-storey model has an L-shaped plan on a six-by-four bay grid, giving a 21.96 m by 14.64 m envelope; the plan is maintained over the full height and therefore contains no setback. The models are three-dimensional, and the bracing was placed in the perimeter frames of each plan.

Five vertical brace-placement layouts were considered for each bracing type. These comprised bracing at every storey (All), one braced storey followed by one unbraced storey (1-1), two braced storeys followed by two unbraced storeys (2-2), two braced storeys followed by one unbraced storey (2-1), and three braced storeys followed by one unbraced storey (3-1). Each sequence was repeated over the building height according to the corresponding layout definition. Altering the sequence of braced and unbraced storeys changes the vertical stiffness distribution of the frame and consequently affects lateral deformation, inter-storey drift, and the forces transferred among the structural members.

The combination of three storey numbers, three bracing types, and five brace-placement layouts resulted in 45 structural configurations. For each height group, the same modelling, loading, and optimization procedures were applied to the alternative brace types and layouts, providing a consistent basis for comparing their resulting best-observed feasible weights. The 45 configurations form a complete and balanced factorial design. Each bracing type and each storey number is represented by 15 configurations and each brace-placement layout by nine, so that the main effects of the three variables are mutually orthogonal and can be estimated independently of one another. For the reference Ridge model the effective number of parameters, obtained as the trace of the corresponding hat matrix, is 7.94, which corresponds to approximately 5.7 observations per estimated parameter.

#### 2.1.2. Design Variables and Optimization Objective

**Figure 1:**
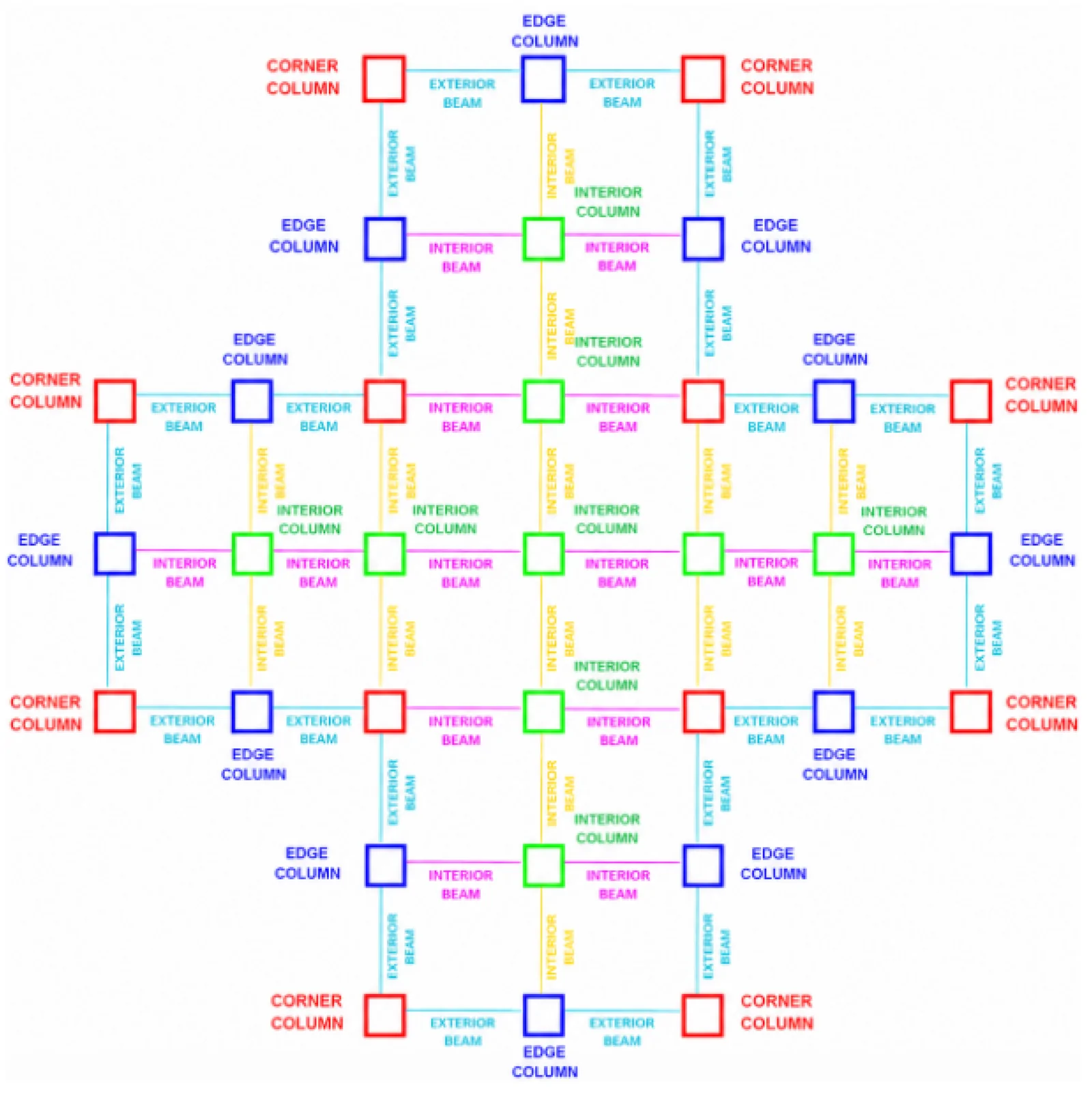
Grouping of structural members adopted for the optimization.

The design variables were the cross-sections assigned to the beams, columns, and bracing members. Assigning an independent section to every individual member would considerably increase the dimensionality of the search space and the computational cost of the optimization. Members with comparable structural functions and locations were therefore collected into design groups, and all members belonging to the same group were assigned an identical section during each optimization evaluation. The grouping was defined according to member location and structural role so that the number of independent design variables could be reduced while maintaining a practical distribution of sections throughout the frame. The adopted member-grouping scheme is shown in Figure 1.

Section selection was restricted to standard steel sections available in the AISC Shapes Database v15.0 associated with the 15th Edition Steel Construction Manual. W-shaped sections were considered for beams and columns, while square and rectangular HSS sections were used for the bracing members. To control the dimensionality of the optimization problem, structural members were grouped according to their function and vertical location. Six section groups were defined within each vertical zone: corner columns, edge columns, interior columns, exterior beams, interior beams, and braces. A common set of section variables was assigned over three consecutive storeys, with the uppermost two storeys forming the final zone in the 17-storey model. Accordingly, the 17-, 24-, and 30-storey models contained 36, 48, and 60 independent section-design variables, respectively. All members belonging to the same design group were assigned an identical section during a candidate evaluation.

The grouping restricts the design space. Any section arrangement that the grouped formulation can represent can also be represented when the members are sized individually, so the weights obtained here are upper bounds on those attainable with a finer parameterization. The restriction is substantial: the 17-storey model has 33 columns in each of its lower eight storeys and nine in each storey above, a total of 345 column members, which the grouping represents by 18 column variables. It also corresponds to the way columns are supplied, since columns in multi-storey steel frames are fabricated and erected in multi-storey lengths and spliced between floors; a three-storey zone is 9.0 m in the 17- and 24-storey models and 11.0 m in the 30-storey model. Assigning an individual section to every member would give lighter designs, but would produce a different section at almost every storey and on every column line, and therefore a larger number of distinct fabrication items and splice details.

Each design variable represented a discrete section selected from the prescribed AISC section database. The optimization algorithms therefore searched among discrete section alternatives rather than treating the cross-sectional properties as unrestricted continuous variables. W-shaped beam and column sections and HSS bracing sections were selected directly from the corresponding section lists stored in the SAP2000 model. The selected sections were transferred to the associated structural-member groups through the OAPI before each structural analysis.

**Figure 2:**
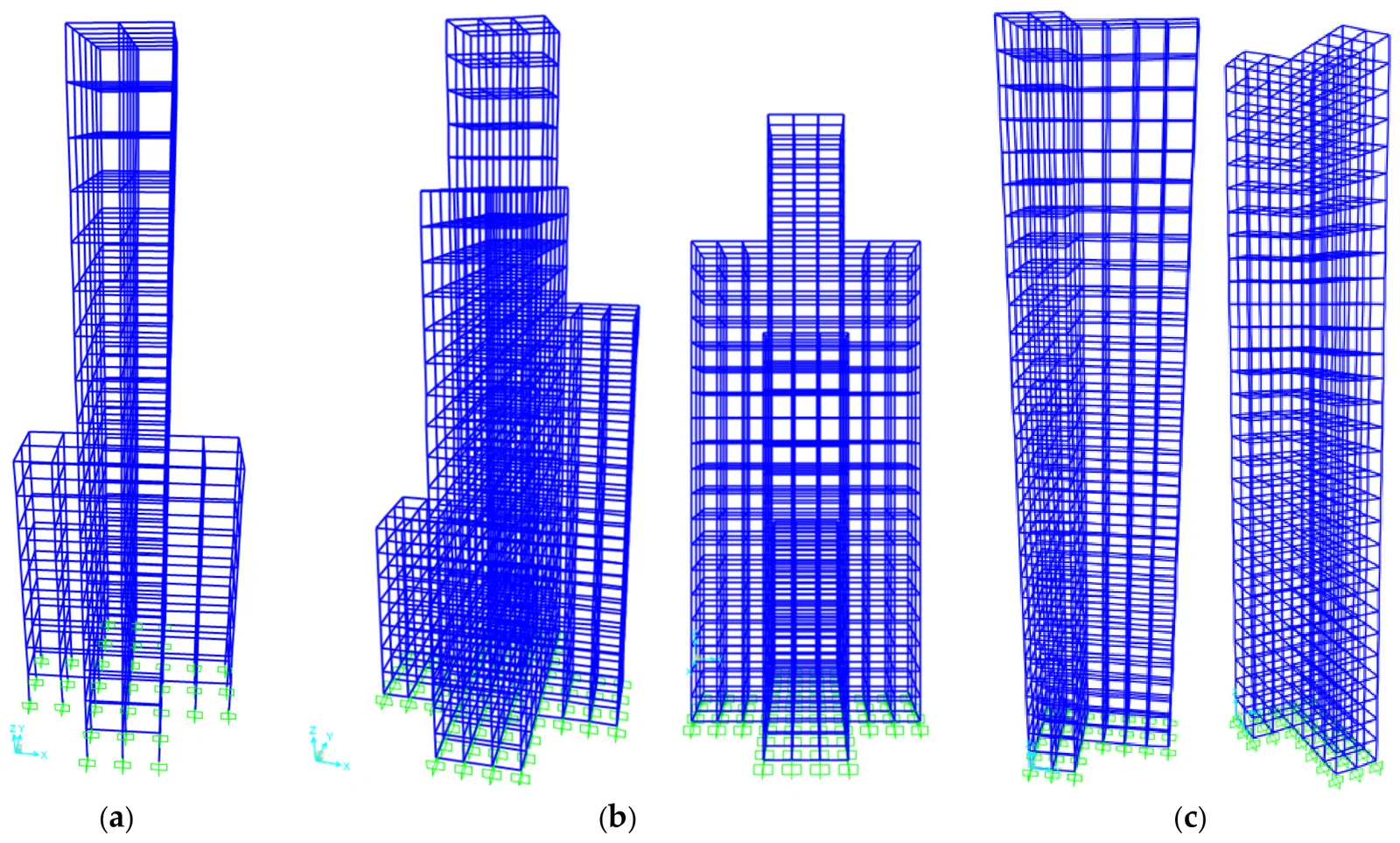
Structural configurations used in the study: (**a**) 17-storey, (**b**) 24-storey, and (**c**) 30-storey frame models. Blue lines denote the frame members and green symbols the fixed supports.

The 17-, 24-, and 30-storey frame models together with the V-, X-, and K-bracing arrangements considered in the analyses are illustrated in Figure 2.

For each candidate solution generated by an optimization algorithm, the selected sections were assigned to the corresponding member groups in SAP2000 through the OAPI. The structural model was then analysed, the required response quantities were returned to MATLAB, and the candidate design was checked against the prescribed strength, displacement, drift, and geometric compatibility constraints. The resulting fitness value was subsequently returned to the optimization algorithm, and this SAP2000–MATLAB analysis cycle was repeated throughout the search.

The objective of the optimization was to minimize the total structural weight of the steel frame. The objective function is defined by Equation (1), in which the contribution of each member is calculated from the unit weight of steel, member length, and assigned cross-sectional area.

(1) $$W=\sum_{i=1}^{q}\gamma_{i}L_{i}x_{i}$$

where *W* denotes the total structural weight, *γ_i_* denotes the unit weight of steel associated with the *i*th design term, *L_i_* is the total member length represented by that term, *x_i_* is the cross-sectional area assigned to the corresponding design variable, and *q* is the total number of design variables. Thus, when a single design variable represents several structural members within the same group, the contribution of all members assigned to that group is included in the calculation of *W*. Equation (1) was evaluated for every candidate design generated during the optimization process.

The structural models were analysed under the gravity and lateral loading conditions adopted for each height group. Dead load *D* was taken as 2.89 kN/m^2^ for both typical floors and the roof, whereas live load *L* was taken as 2.39 kN/m^2^ for typical floors and was not applied at roof level. Snow load *S* was not applied to typical floors and was taken as 0.7543 kN/m^2^ at roof level [51]. These gravity-loading assumptions were maintained for all brace types and layouts within the corresponding structural models.

Wind loading was additionally considered for the 24- and 30-storey models in accordance with ASCE 7-16 [53], using a basic wind speed of 85 mph (38 m/s). Wind effects were incorporated through the load combinations 1.2D + 1.0WY + 1.0L + 0.5S and 1.2D + 1.0WX + 1.0L + 0.5S, where WX and WY denote wind actions in the global X- and Y-directions, respectively. Wind loading was not included in the 17-storey models under the loading scheme adopted in this study. The same loading definition was maintained for all bracing types and brace-placement layouts belonging to a given height group. Consequently, comparisons among bracing alternatives within the same height group were made under identical loading conditions, whereas comparisons between different height groups also reflect the difference in the adopted wind-loading treatment.

Seismic actions were not included in the load combinations. The optimization problem was formulated with the AISC-LRFD [54] strength and serviceability requirements described in Section 2.1.3 under the gravity and wind combinations given above, that is, on a non-seismic design basis. Adding earthquake actions would not simply introduce one further load case into this formulation. Concentrically braced frames intended to resist earthquake actions are governed in addition by the capacity-design requirements of AISC 341 [55], which place local and global slenderness limits on the braces, require beams and columns to be proportioned for the forces delivered by the braces at their expected yield and post-buckling strengths, and restrict the admissible brace arrangements. K-braced frames, in which the two diagonals meet the column between floor levels, are not permitted as special concentrically braced frames, because buckling of the compression diagonal then applies an unbalanced transverse force to the column. The constraint set and the set of configurations available for comparison would therefore both differ, and the results reported in this study apply to the gravity- and wind-governed conditions defined above.

#### 2.1.3. Design Constraints

Minimizing structural weight alone does not result in an acceptable steel-frame design because progressive reductions in member size may lead to inadequate strength, excessive displacement or inter-storey drift, or impractical combinations of connected member sections. The optimization was therefore formulated as a constrained minimum-weight problem in accordance with the AISC-LRFD [54] provisions adopted in the structural analyses. Comparable constrained formulations have been employed in previous minimum-weight optimization studies of steel frames [56].

For members subjected to combined axial compression and biaxial bending, the member interaction requirements were expressed by Equations (2) and (3):

(2) $$g_{1}\left(x\right)=\left(\frac{P_{u}}{\phi_{c}P_{n}}\right)+\frac{8}{9}\left(\frac{M_{\mathrm{ux}}}{\phi_{b}M_{\mathrm{nx}}}+\frac{M_{\mathrm{uy}}}{\phi_{b}M_{\mathrm{ny}}}\right)-1.0\leq 0\;\;\;\;\frac{P_{u}}{\phi_{c}P_{n}}\geq 0.2$$

(3) $$g_{2}\left(x\right)=\left(\frac{P_{u}}{2\phi_{c}P_{n}}\right)+\left(\frac{M_{\mathrm{ux}}}{\phi_{b}M_{\mathrm{nx}}}+\frac{M_{\mathrm{uy}}}{\phi_{b}M_{\mathrm{ny}}}\right)-1.0\leq 0\;\;\;\;\frac{P_{u}}{\phi_{c}P_{n}}<0.2$$

In Equations (2) and (3), *P_u_* is the required axial compressive strength and *P_n_* is the nominal axial compressive strength. *M_ux_* and *M_uy_* denote the required flexural strengths about the major and minor axes, respectively, while *M_nx_* and *M_ny_* are the corresponding nominal flexural strengths. The resistance factors for compression and flexure are represented by *ϕ_c_* and *ϕ_b_*, respectively. Equation (2) applies when the axial utilization ratio *P_u_*(*ϕ_c_P_n_*) is equal to or greater than 0.2, whereas Equation (3) applies when this ratio is less than 0.2. Both expressions were required to satisfy *g*(*x*) ≤0 for the corresponding member to be considered feasible.

The shear-strength constraint was expressed in demand-to-capacity form as

(4) $$g_{3}(x)=\frac{V_{u}}{{\phi_{v}V}_{n}}-1\leq 0$$

where *V_u_* is the required shear strength, *V_n_* is the nominal shear strength, and *ϕ_v_* is the resistance factor for shear. Equation (4) therefore requires the factored shear demand to remain within the available design shear strength ${\phi_{v}V}_{n}$.

Serviceability requirements were imposed in addition to the strength constraints. The displacement constraint was defined by Equation (5):

(5) $$g_{4}\left(x\right)=\frac{\delta_{i}}{\delta_{\mathrm{iu}}}-1\leq 0\;\;\;\;i=1,\ldots ,n_{d}$$

where *δ_i_* is the displacement or deflection associated with the *i*th controlled response, *δ_iu_* is its corresponding allowable value, and *n*_d_ denotes the total number of displacement constraints. For beams supporting plaster or other brittle finishes, the allowable beam deflection was taken as *L*/300, where *L* is the corresponding beam span. Equation (5) ensures that each controlled displacement remains below its prescribed serviceability limit.

Inter-storey drift was checked separately because satisfaction of the overall displacement requirement does not necessarily prevent excessive relative movement between adjacent floors. The inter-storey drift constraint was defined as

(6) $$g_{5}\left(x\right)=\frac{\left(\delta_{j}-\delta_{j-1}\right)}{h_{j}\delta_{\mathrm{ju}}}-1\leq 0\;\;\;\;j=1,\ldots ,n_{s}$$

where *δ_j_* and *δ_j_*_−1_ are the lateral displacements of the *j*th and (*j* − 1)th storeys, respectively, *h_j_* is the height of the *j*th storey, *δ_ju_* is the allowable inter-storey drift ratio, and *n*_s_ is the total number of storeys. The allowable drift was taken as *h_j_*/300; accordingly, the limiting drift ratio used in Equation (6) was *δ_ju_* = 1/300.

Geometric compatibility was also checked at the beam–column connections using Equation (7):

(7) $$g_{6}\left(x\right)=\frac{B_{\mathrm{fbj}}}{B_{\mathrm{fcj}}}-1\leq 0,\;\;\;\;\frac{B_{\mathrm{fbj}}}{D_{\mathrm{sj}}-2t_{\mathrm{cj}}}-1\leq 0\;\;\;\;j=1,2,\ldots ,n_{j}$$

where *B_fbj_* is the beam flange width at the *j*th beam–column joint, *B_fcj_* is the flange width of the corresponding column, *D_sj_* is the depth of the column section, *t_cj_* is the column flange thickness, and *n*_j_ is the number of beam–column joints subjected to these compatibility checks. The first condition in Equation (7) prevents the beam flange width from exceeding the column flange width, while the second restricts the beam flange width relative to the clear depth between the column flanges. These conditions prevent the optimization process from accepting section combinations that satisfy the weight objective but are geometrically incompatible at the connection.

Column sections were additionally constrained to maintain a practical progression over the building height. The section depth at an upper storey was not permitted to exceed the depth of the corresponding column section immediately below:

(8) $$g_{7}(x)=\frac{D_{s+1}}{D_{s}}-1\leq 0$$

Similarly, the unit mass of the selected column section was required to remain non-increasing with height:

(9) $$g_{8}\left(x\right)=\frac{m_{s+1}}{m_{s}}-1\leq 0$$

The range over which the adjacent-storey continuity constraints in Equations (8) and (9) were evaluated is defined by

*s* = 1,…,*n*_u_ (10)

where *D_s_* and *D_s_*_+1_ are the depths of corresponding column sections at consecutive storeys *s* and *s* + 1, respectively, and *m_s_* and *m_s_*_+1_ are their corresponding unit masses. The parameter *n*_u_ denotes the number of adjacent-storey column comparisons included in these continuity checks. Equations (8) and (9) therefore prevent increases in column depth or unit mass towards the upper storeys, while Equation (10) defines the index range over which these requirements are imposed.

Taken together, Equations (1)–(10) define the constrained minimum-weight design problem considered in this study. Equation (1) defines the objective function; Equations (2) and (3) control combined axial-force and flexural utilization; Equation (4) imposes the shear-strength requirement; Equations (5) and (6) control member displacement and inter-storey drift; and Equations (7)–(10) impose connection compatibility and column-continuity requirements. Candidate designs violating one or more of these constraints were treated as infeasible and were handled using the penalty-function procedure described in Section 2.2.5.

Stability was addressed by the direct analysis method of Chapter C of AISC 360-16 [54]. A general second-order analysis was carried out, the prescribed stiffness reduction was applied, and an allowance was made for initial geometric imperfections, so that the effective length factor used in the calculation of member compressive strengths is unity.

The nominal strengths appearing in Equations (2)–(4) were not computed independently. After each structural analysis the SAP2000 steel frame design module was executed, and the member strengths were obtained from it in accordance with AISC 360-16 [54] using the LRFD provisions. The checks applied are therefore those of the specification: classification of the cross-section against the width-to-thickness limits of Section B4, with compact, noncompact and slender elements treated by the corresponding local-buckling provisions; tensile strength from Chapter D; compressive strength from Chapter E; flexural strength from Chapter F, including lateral-torsional buckling; shear strength from Chapter G; and the combined-force interaction of Chapter H. The candidate section list was not restricted to compact profiles, and a section was retained only if it satisfied the applicable strength and stability requirements. Because the braces were released for bending at both ends, they were designed as axially loaded members carrying both tension and compression, and their effective slenderness ratio was limited to 200 as an additional design criterion. No composite floor action was assumed for the beams, so the laterally unbraced length was taken as the full span where no intermediate lateral restraint was present, and as the distance between restraint points elsewhere. The member forces, displacements and demand-to-capacity ratios were returned to MATLAB through the OAPI, where the constraint functions of Equations (2)–(10) were evaluated and the penalty procedure of Section 2.2.5 was applied. The optimization therefore searched the discrete section space, while the strength assessment of every candidate design remained that of AISC 360-16.

### 2.2. Metaheuristic Optimization Framework

Metaheuristic algorithms are well suited to structural weight optimization problems in which the design variables are discrete and the feasible region is governed by several interacting strength, serviceability, and geometric constraints. Unlike gradient-based methods, these algorithms do not require derivatives of the objective or constraint functions and can therefore be applied directly to section-selection problems involving discrete steel profiles [57]. Their use is particularly appropriate in the present study because each candidate design requires the assignment of member sections, structural analysis in SAP2000, evaluation of the constraints defined in Section 2.1.3, and subsequent updating of the candidate solution according to the search mechanism of the optimization algorithm.

Four population-based algorithms were employed: Particle Swarm Optimization (PSO), Grey Wolf Optimization (GWO), the Honey Badger Algorithm (HBA), and the Aquila Optimizer (AO). PSO and GWO were included as established algorithms that have been applied extensively to structural and engineering optimization problems, whereas HBA and AO were included as more recently proposed algorithms with substantially different exploration and exploitation mechanisms. Using algorithms with different search strategies was intended to reduce dependence of the final design on the behaviour of a single optimization method and to provide a broader search of the discrete section space.

For each of the 45 structural configurations, all four algorithms were applied to the same minimum-weight design problem and were subjected to the same structural constraints. Each candidate solution generated by an algorithm represented a set of steel sections assigned to the member groups defined in Section 2.1.2. The corresponding sections were transferred from MATLAB to SAP2000 through the OAPI, after which the structural analysis was performed and the resulting member forces, displacements, and inter-storey drifts were returned to MATLAB. The candidate solution was then evaluated using the structural weight defined by Equation (1) together with the strength, serviceability, and compatibility constraints given in Equations (2)–(10). Designs violating these requirements were handled through the penalty-function procedure described in Section 2.2.5.

Because PSO, GWO, HBA, and AO are stochastic search algorithms, the result obtained from an individual search cannot be interpreted as proof of a global optimum. Accordingly, for each structural configuration, the lowest feasible structural weight obtained among the four algorithm-specific searches was retained as the best-observed feasible weight and used as the target value in the subsequent machine-learning analysis. This procedure resulted in 180 optimization runs in total, corresponding to 45 structural configurations analysed using four algorithms. The use of the lowest feasible result from the four searches reduces the dependence of the generated dataset on the convergence behaviour of any single algorithm, although it does not remove the stochastic nature of the optimization process. For clarity, the term best-observed feasible weight is used throughout the paper to denote the lowest constraint-satisfying weight identified among the four algorithm-specific searches carried out for a given configuration. It is a feasible solution of the constrained minimum-weight design problem defined by Equations (1)–(10), but it is not presented as a proven global optimum.

The mathematical formulations and updating procedures of HBA, AO, GWO, and PSO are presented in Section 2.2.1, Section 2.2.2, Section 2.2.3 and Section 2.2.4, respectively. The common constraint-handling procedure, stopping conditions, population size, iteration limit, and algorithm-specific search parameters are described in Section 2.2.5 and summarized in Table 2.

#### 2.2.1. Honey Badger Optimization Algorithm

The Honey Badger Algorithm (HBA), introduced by Hashim et al. [58], is a population-based metaheuristic inspired by the foraging behaviour of honey badgers. Its search procedure alternates between two updating mechanisms, referred to as the digging and honey phases. The digging phase combines movement towards the best solution found so far with intensity-dependent and oscillatory components, whereas the honey phase provides a more direct movement towards that solution. In the present application, each honey badger represents a candidate vector containing the design variables associated with the structural member groups.

For a population of *N* candidate solutions and a problem dimension of *Dim*, the population is represented by Equation (11):

(11) $$X\;=\left[\begin{array}{ccccc} x_{1,1} & \cdots & x_{1,j} & x_{1,\;\mathrm{Dim}-1\;} & x_{1,\;\mathrm{Dim}\;}\\ x_{2,1} & \cdots & x_{2,j} & \cdots & x_{2,\;\mathrm{Dim}\;}\\ \cdots & \cdots & x_{i,j} & \cdots & \cdots \\ \vdots & \vdots & \vdots & \vdots & \vdots \\ x_{N-1,1} & \cdots & x_{N-1,j} & \cdots & x_{N-1,\mathrm{Dim}}\\ x_{N,1} & \cdots & x_{N,j} & x_{N,\mathrm{Dim}-1} & x_{N,\mathrm{Dim}} \end{array}\right]$$

where *x_i_*_,*j*_ denotes the *j*th design variable of the *i*th candidate solution. The design vector of the *i*th candidate is therefore expressed by Equation (12):

(12) $$x_{i}=\left[x_{i}^{1},x_{i}^{2},\ldots ,x_{i}^{\mathrm{Dim}}\right]$$

The initial value of each component is generated within its prescribed search bounds using Equation (13):

*x_i_* = *lb_i_* + *r*_1_ ∗ (*ub_i_* − *lb_i_*) (13)

where *lb_i_* and *ub_i_* are the lower and upper bounds of the design variables, respectively, and *r*_1_ is a uniformly distributed random number in [0, 1].

HBA controls movement towards the best solution through an intensity term. The intensity associated with the *i*th candidate is calculated using Equation (14):

(14) $$I_{i}=r_{2}*\frac{S}{4\pi d_{i}^{2}}$$

where *r*_2_ is a uniformly distributed random number. The source-strength term *S* is evaluated from Equation (15):

(15) $$S={\left(x_{i}-x_{i+1}\right)}^{2}$$

and the distance between the *i*th candidate and the best solution found so far is determined from Equation (16):

*d_i_* = *x_prey_* − *x_i_* (16)

where *x_prey_* denotes the current best solution. The operations in Equations (14)–(16) are evaluated component-wise for the design vector.

The density factor *α*, which progressively reduces the extent of exploratory movement as the search proceeds, is defined by Equation (17):

(17) $$\alpha =C*\exp\left(\frac{-t}{t_{\max}}\right)$$

where *t* is the current iteration, *t_max_* is the maximum number of iterations, and *C* is a control parameter. In the present study, *C* = 2, as reported in Table 2.

During the digging phase, the position of the *i*th candidate is updated according to Equation (18):

*x_new_* = *x_prey_* + *F* ∗ *β* ∗ *I* ∗ *x_prey_* + *F* ∗ *r*_3_ ∗ *α* ∗ *d_i_* ∗ |cos(2*πr*_4_) ∗ [1 − cos(2*πr*_5_)]| (18)

where *β* controls the ability of the algorithm to move towards promising regions and was set to 6 in this study. The random variables *r*_3_, *r*_4_, and *r*_5_ are independently sampled from [0, 1]. The direction flag *F*, which allows the search direction to change during the updating process, is determined using Equation (19):

(19) $$F=\{\begin{array}{cc} \;\;\;\;\;\;\;\;\;\;1, & r_{6}<0.5\\ \;-1, & \;\;\;\;\;\mathrm{otherwise} \end{array}$$

where *r*_6_ is another uniformly distributed random number in [0, 1].

In the honey phase, the update is simplified to a direct movement towards the current best solution, as given by Equation (20):

*x_new_* = *x_prey_* + *F* ∗ *r*_7_ ∗ *α* ∗ *d_i_* (20)

where *r*_7_ is uniformly distributed in [0, 1]. Equations (18)–(20) therefore provide two distinct search behaviours: the digging phase introduces additional intensity and oscillatory terms to explore alternative regions, whereas the honey phase increasingly concentrates the search around the best solution as *α* decreases.

#### 2.2.2. Aquila Optimization Algorithm

The Aquila Optimizer (AO), proposed by Abualigah et al. [59], is a population-based metaheuristic in which candidate solutions are updated through four search strategies representing successive stages of exploration and exploitation. In the present structural optimization problem, an Aquila position represents a candidate set of design variables assigned to the member groups.

The population of *N* candidate solutions is expressed by Equation (21):

(21) $$X=\left[\begin{array}{ccccc} x_{1,1} & \cdots & x_{1,j} & x_{1,\;\mathrm{Dim}-1\;} & x_{1,\;\mathrm{Dim}\;}\\ x_{2,1} & \cdots & x_{2,j} & \cdots & x_{2,\;\mathrm{Dim}\;}\\ \cdots & \cdots & x_{i,j} & \cdots & \cdots \\ \vdots & \vdots & \vdots & \vdots & \vdots \\ x_{N-1,1} & \cdots & x_{N-1,j} & \cdots & x_{N-1,\mathrm{Dim}}\\ x_{N,1} & \cdots & x_{N,j} & x_{N,\mathrm{Dim}-1} & x_{N,\mathrm{Dim}} \end{array}\right]$$

where *N* is the population size and *Dim* is the number of design variables. The *j*th component of the *i*th candidate is initialized within the corresponding search bounds according to Equation (22):

*X_ij_* = *rand* × (*UB_j_* − *LB_j_*) + *LB_j_*,      *i* = 1,2,…,*Nj* = 1,2,…, *Dim* (22)

where *UB_j_* and *LB_j_* are the upper and lower bounds of the *j*th variable, respectively, and *rand* denotes a newly generated uniform random number in [0, 1].

The first AO strategy performs broad exploration using the current best solution and the mean position of the population. The corresponding update is given by Equation (23):

(23) $$X_{1}(t+1)=X_{\mathrm{best}\;}(t)\times \left(1-\frac{t}{T}\right)+\left(X_{M}(t)-X_{\mathrm{best}}(t)\;\mathrm{rand}\right)$$

where *X_best_*(*t*) is the best solution found up to iteration *t*, *T* is the maximum number of iterations, and *X_M_*(*t*) is the mean population position. The latter is calculated from Equation (24):

(24) $$X_{M}(t)=\frac{1}{N}\sum_{i=1}^{N}\;X_{i}(t),\forall j=1,2,\ldots ,\mathrm{Dim}$$

with the averaging performed for each of the *Dim* design variables.

The second strategy introduces Lévy-flight-based exploration and a randomly selected population member [59,60]:

*X*_2_(*t* + 1) = *X_best_*(*t*) × *Levy*(*D*) + *X_R_*(*t*) + (*y* − *x*) ∗ *rand* (25)

where *X_R_*(*t*) is a randomly selected candidate from the current population, *D* denotes the search-space dimension, and *Levy*(*D*) represents the Lévy-flight step used to introduce occasional longer search movements. The Lévy-flight term was evaluated according to the standard AO formulation with a Lévy exponent of 1.5. The variables *x* and *y* describe the spiral component of the AO search. In this formulation, the spiral-control constants were *U* = 0.00565 and *ω* = 0.005, as summarized in Table 2 [59,60,61].

After the exploratory stages, AO shifts towards exploitation. The third strategy is expressed by Equation (26):

*X*_3_(*t* + 1) = (*X_best_*(*t*) − *X_M_*(*t*)) ∗ *α* − *rand* + ((*UB* − *LB*) ∗ *rand* + *LB*) ∗ *δ* (26)

where *α* and *δ* are exploitation-adjustment coefficients. Both parameters were set to 0.1 in the present study.

The fourth strategy concentrates the search further around the best solution and is given by Equation (27):

*X*_4_(*t* + 1) = *QF* ∗ *X_best_*(*t*) − (*G*_1_ ∗ *X*(*t*) ∗ *rand*) − *G*_2_ ∗ *Levy*(*D*) + *rand* ∗ *G*_1_ (27)

where *X*(*t*) is the current candidate position, *QF* is the quality function, and *G*_1_ and *G*_2_ are control parameters governing movement magnitude and the progressive reduction in the search range. These quantities are determined from Equations (28)–(30):

(28) $$\mathrm{QF}(t)=t^{\frac{2*\mathrm{rand}-1}{{\left(1-T\right)}^{2}}}$$

*G*_1_ = 2 ∗ *rand* − 1 (29)

(30) $$G_{2}=2*\left(1-\frac{t}{T}\right)$$

Equation (29) allows *G*_1_ to vary within [−1, 1], whereas Equation (30) decreases *G*_2_ from approximately 2 towards 0 as the iteration number approaches *T*. Consequently, Equations (23)–(25) provide the principal exploratory movements of AO, while Equations (26)–(30) progressively concentrate the search around promising candidate designs.

#### 2.2.3. Grey Wolf Optimization Algorithm

The Grey Wolf Optimizer (GWO), introduced by Mirjalili et al. [62], represents candidate solutions through a hierarchical population in which the three best solutions are denoted by *α*, *β*, and *δ*. The remaining candidates are guided by these three solutions during the search. GWO has also been successfully adapted to steel structural optimization problems involving discrete member selection [63].

The basic encircling mechanism is defined by Equations (31) and (32):

*D* = |*C* ∗ *X_p_*(*t*) − *X*(*t*)| (31)

*X*(*t* + 1) = *X_p_*(*t*) − *AD* (32)

where *X*(*t*) is the current candidate position, *X_p_*(*t*) denotes the position of the target solution, and *D* represents the distance measure used in the updating process. The coefficient vectors *A* and *C* are calculated using Equations (33) and (34):

*A* = *a*(2 ∗ *r*_1_ − 1) (33)

*C* = 2 ∗ *r*_2_ (34)

where *r*_1_ and *r*_2_ are independently generated uniform random vectors in [0, 1]. The convergence parameter *a* decreases linearly from 2 to 0 during the optimization, progressively reducing the magnitude of the exploratory movements.

Rather than following a single target solution, GWO uses the alpha, beta, and delta candidates simultaneously. Their respective distance vectors are calculated using Equation (35):

(35) $$\begin{array}{c} D_{\alpha }=\left|C_{1}*X_{\alpha }-X(t)\right|\\ D_{\beta }=\left|C_{2}*X_{\beta }-X(t)\right|\\ D_{\delta }=\left|C_{3}*X_{\delta }-X(t)\right| \end{array}$$

where *X_α_*, *X_β_* and *X_δ_* are the positions of the three best candidate solutions. The corresponding intermediate positions are then determined from Equation (36):

(36) $$\begin{array}{c} X_{1}=X_{\alpha }-A_{1}D_{\alpha }\\ X_{2}=X_{\beta }-A_{2}D_{\beta }\\ X_{3}=X_{\delta }-A_{3}D_{\delta } \end{array}$$

where *A*_1_, *A*_2_, and *A*_3_, together with *C*_1_, *C*_2_, and *C*_3_ in Equation (35), are independently generated using Equations (33) and (34). The updated position is finally obtained from the average influence of the three leader solutions:

(37) $$X(t+1)=\frac{X_{1}+X_{2}+X_{3}}{3}$$

Thus, Equation (37) combines information from the three best solutions rather than forcing the population to follow a single candidate, while the decreasing parameter *a* controls the gradual transition from wider exploration to more localized exploitation.

#### 2.2.4. Particle Swarm Optimization Algorithm

Particle Swarm Optimization (PSO) was originally introduced by Eberhart and Kennedy [64]. In PSO, each particle represents a candidate structural design whose position corresponds to the current design-variable vector. The movement of each particle is influenced by its current velocity, its own best position found during the search, and the best position identified by the swarm.

The initial particle position is generated within the prescribed design-variable bounds using Equation (38):

(38) $$x_{0}^{i}=x_{\min}+r\left(x_{\max}-x_{\min}\right)$$

where $x_{0}^{i}$ is the initial position of particle *i*, *x_min_* and *x_max_* denote the lower and upper search bounds, respectively, and *r* is uniformly distributed in [0, 1]. The corresponding initial velocity is defined by Equation (39):

(39) $$v_{0}^{i}=\left[\frac{\left(x_{\min}+r\left(x_{\max}-x_{\min}\right)\right)}{\Delta t}\right]$$

where $v_{0}^{i}$ is the initial velocity and Δ*t* denotes the numerical step used in the position update.

At each iteration, the structural fitness of every particle is first evaluated. The personal best position $p_{k}^{i}$ of particle *i* and the global best position $p_{k}^{g}$ of the swarm are then updated. The particle velocity and position are subsequently calculated together using Equation (40):

(40) $$\begin{array}{c} v_{k+1}^{i}=\omega v_{k}^{i}+c_{1}r_{1}(p_{k}^{i}-x_{k}^{i})+c_{2}r_{2}(p_{k}^{g}-x_{k}^{i})\\ x_{k+1}^{i}=x_{k}^{i}+v_{k+1}^{i}\Delta t \end{array}$$

where $x_{k}^{i}$ and $v_{k}^{i}$ are the position and velocity of particle *i* at iteration *k*, respectively; *r*_1_ and *r*_2_ are independent random numbers in [0, 1]; *ω* is the inertia weight; and *c*_1_ and *c*_2_ are the cognitive and social acceleration coefficients. In the present study, *ω* = 1 and *c*_1_ = *c*_2_ = 2, as given in Table 2. The first line of Equation (40) updates the search direction using the particle’s own search history and the swarm-level best solution, while the second line advances the particle to its new position.

#### 2.2.5. Constraint Handling Using Penalty Function

The four algorithms described above are unconstrained search procedures in their basic forms, whereas the present steel-frame optimization problem is governed by the strength, serviceability, geometric compatibility, and column-continuity requirements defined in Equations (2)–(10). A penalty-function approach was therefore used to incorporate violations of these structural requirements into the fitness evaluation [65].

For each candidate design, the total constraint violation *V* was calculated using Equation (41):

(41) $$V=\sum_{i=1}^{\mathrm{nc}}\;C_{i};C_{i}=\left\{\begin{array}{l} 0\;\mathrm{for}\;g_{i}(x)\leq 0\\ g_{i}(x)\;\mathrm{for}\;g_{i}(x)>0 \end{array}\right\}$$

where *nc* is the total number of evaluated constraints, *g_i_*(*x*) is the normalized form of the *i*th constraint, and *C_i_* is its positive violation contribution. A feasible candidate therefore has *V* = 0, whereas any violation of the constraints produces *V* > 0.

The penalized objective function used by all four algorithms was then calculated from Equation (42):

*φ* = *W* ⋅ (1 + *V*)^2^ (42)

where *W* is the structural weight calculated from Equation (1) and *φ* is the fitness value supplied to the optimization algorithm. When all constraints are satisfied, *V* = 0 and Equation (42) reduces to *φ* = *W*. For an infeasible design, the multiplicative term (1 + *V*)^2^ increases the fitness value according to the accumulated constraint violation and therefore reduces the likelihood that the design will be retained during the subsequent search.

The multiplicative form was adopted because the penalty then scales with the structural weight of the candidate design. The weights returned by the searches in this study range from about 2260 to 16,910 kN, and a given relative constraint violation therefore carries the same relative cost at every height, whereas an additive penalty would require a coefficient calibrated separately for each height group. The exponent controls only how far into the infeasible region the search is allowed to look: with the exponent set to two, an infeasible design can outrank a feasible one only if it is lighter by at least 2.0% for a 1% total violation, 9.3% for a 5% violation and 17.4% for a 10% violation; the corresponding figures are 1.0%, 4.8% and 9.1% for an exponent of one, and 2.9%, 13.6% and 24.9% for an exponent of three. The value of two is therefore intermediate among the exponents in common use. Between two feasible designs the penalty term is unity for both, so the exponent cannot affect which feasible design is preferred.

The same objective function, constraint definitions, penalty formulation, population size, and maximum iteration limit were used for HBA, AO, GWO, and PSO so that the algorithms were compared under a common structural-design problem and computational budget. Algorithm-specific parameters followed the settings associated with the cited formulations [58,59,62,64]. A population size of 30 and a maximum of 50,000 iterations were adopted for every optimization run. The complete set of search parameters is summarized in Table 2.

For each iteration, MATLAB generated or updated the candidate design vectors according to the relevant algorithm, transferred the corresponding section assignments to SAP2000 through the OAPI, initiated the structural analysis, retrieved the required structural-response quantities, evaluated Equations (1)–(10), and calculated the penalized fitness using Equations (41) and (42). This cycle continued until the maximum iteration limit was reached. Each of the four algorithms was applied once to each of the 45 structural configurations, resulting in 180 optimization runs. Accordingly, the algorithm comparisons reported in Section 3 describe the outcomes of these individual stochastic searches rather than distributions obtained from repeated runs of the same algorithm. For each structural configuration, the lowest feasible weight among the four algorithm-specific results was retained as the best-observed feasible weight for subsequent machine-learning analysis.

### 2.3. Machine Learning Model Development and Evaluation Procedure

The optimization stage produced 45 structural configurations defined by three storey numbers (17, 24, and 30), three bracing types (V, X, and K), and five brace-placement layouts. Each configuration was therefore represented by three input variables: storey number, bracing type, and brace-placement layout. The response variable was the lowest feasible structural weight obtained from the four algorithm-specific searches described in Section 2.2. Because the metaheuristic procedures are stochastic and each algorithm was run once for each configuration, this response is referred to hereafter as the best-observed feasible weight rather than a proven global optimum. The storey-number variable takes only three values, and, as described in Section 2.1.1, each of them identifies a different structural system rather than a point on a continuous height scale. It therefore acts as a group indicator. Coding it as a three-level factor instead of a numerical variable changes the mean cross-validated *R*^2^ from 0.904 to 0.893, so the numerical coding carries no information beyond this grouping.

Before model fitting, the two categorical predictors, bracing type and brace-placement layout, were converted to numerical form by one-hot encoding [66], while storey number was standardized to prevent its numerical scale from dominating algorithms that are sensitive to feature magnitude. These pre-processing operations were incorporated into the modelling pipeline so that the same transformations were applied consistently during model fitting and prediction. The resulting dataset was used to compare six regression algorithms representing linear, tree-ensemble, boosting, and distance-based modelling strategies. The purpose of considering models with different levels of flexibility was to determine whether the relationship between structural configuration and best-observed feasible weight could be represented adequately by a relatively simple additive model or whether more flexible nonlinear learners provided a meaningful predictive advantage.

Ridge regression was included as the regularized linear model. Ridge extends ordinary least-squares regression by adding an L_2_ penalty to the coefficient-estimation problem, thereby limiting excessively large coefficients and reducing model variance when the available dataset is small [67]. Its inclusion was particularly useful in the present study because it provides an interpretable reference against which the additional flexibility of the tree-based and distance-based algorithms can be assessed.

Random Forest regression constructs an ensemble of decision trees using bootstrap samples of the training observations and random subsets of predictor variables during tree construction [68]. The individual tree predictions are averaged to reduce the variance associated with a single decision tree. Extremely Randomized Trees (Extra Trees) uses a related ensemble principle but introduces additional randomization in the selection of splitting variables and split thresholds [69]. This distinction provides a useful comparison between two tree-ensemble approaches with different degrees of randomness.

Adaptive Boosting (AdaBoost) builds a sequence of weak regression learners in which subsequent learners place greater emphasis on observations that were represented poorly by the preceding learners [70]. The final estimate is obtained by combining the successive learners. Extreme Gradient Boosting (XGBoost) likewise develops an additive sequence of trees, but each new tree is fitted to reduce the residual error of the existing ensemble while regularization is used to control model complexity [71]. These two algorithms were included to examine whether sequentially corrected nonlinear models could capture configuration-dependent variations that were not represented adequately by a linear relationship.

K-Nearest Neighbours (KNN) was used as a non-parametric, distance-based alternative [72]. For a new configuration, KNN estimates the target from neighbouring observations in the transformed feature space, with closer observations contributing more strongly to the prediction. Its behaviour therefore depends directly on the local distribution of the available configurations and provides a contrasting modelling strategy to the coefficient-based and tree-based approaches.

Model evaluation was performed using both a fixed hold-out partition and five-fold cross-validation. An 80/20 train–test split was first generated using a fixed random seed of 0, resulting in 36 training cases and nine hold-out cases. The hold-out set was retained for the subsequent design-level prediction and uncertainty assessment. To obtain a less partition-dependent comparison among the six regression algorithms, five-fold cross-validation was additionally performed with shuffled observations and the same random seed. In each fold, approximately four-fifths of the 45 configurations were used for fitting and the remaining fifth for testing, so that every configuration appeared once in a test fold across the complete cross-validation procedure. Training and test performance were recorded separately for each fold, allowing the difference between fitting accuracy and predictive accuracy to be examined rather than relying only on goodness of fit to the training data.

Because the dataset contains a complete factorial combination of the three investigated storey levels, three bracing types, and five layouts, the cross-validation procedure assesses prediction for withheld configurations composed of factor levels already represented elsewhere in the dataset. It should therefore be interpreted as an assessment of predictive performance within the investigated design domain. It does not establish extrapolative accuracy for storey numbers outside 17–30 or for bracing types and brace-placement layouts not represented in the present dataset. The behaviour when a factor level is absent from the training set is examined separately in Section 3.2.

Four performance measures were calculated for both the training and test portions of every cross-validation fold: the coefficient of determination *R*^2^, mean square error (MSE), root mean square error (RMSE), and mean absolute error (MAE) [73]. The coefficient of determination was calculated using Equation (43):

(43) $$R^{2}=1-\;\frac{\sum_{i=1}^{n}{\left(y_{i}-{\hat{y}}_{i}\right)}^{2}}{\sum_{i=1}^{n}{\left(y_{i}-\bar{y}\right)}^{2}}$$

where *y_i_* is the best-observed feasible weight of the *i*th configuration, ${\hat{y}}_{i}$ is the corresponding predicted weight, $\bar{y}$ is the mean observed weight of the evaluated partition, and *n* is the number of observations in that partition. Equation (43) measures predictive agreement relative to prediction by the partition mean; values approaching 1 indicate increasingly close agreement, whereas values below 0 are possible when predictions perform worse than the mean-value reference.

The mean square error was calculated from Equation (44):

(44) $$\mathrm{MSE}=\frac{1}{n}\sum_{i=1}^{n}{\left(y_{i}-{\hat{y}}_{i}\right)}^{2}$$

MSE retains the squared-error scale and therefore assigns greater influence to larger deviations. The corresponding root mean square error was calculated using Equation (45):

(45) $$\mathrm{RMSE}=\sqrt{\frac{1}{n}\sum_{i=1}^{n}{\left(y_{i}-{\hat{y}}_{i}\right)}^{2}}$$

Because Equation (45) returns the error to the original units of the target variable, RMSE is reported in kN and provides a direct indication of the characteristic magnitude of the prediction error while retaining greater sensitivity to relatively large deviations.

The mean absolute error was determined from Equation (46):

(46) $$\mathrm{MAE}=\frac{1}{n}\sum_{i=1}^{n}|y_{i}-{\hat{y}}_{i}|$$

Unlike RMSE, Equation (46) weights all absolute deviations linearly and is therefore less influenced by isolated large errors. The four metrics were considered together because *R*^2^ describes the proportion of variation reproduced by a model, whereas RMSE and MAE quantify prediction error directly in kN and MSE provides the squared-error measure underlying RMSE.

Model selection was based primarily on the five-fold cross-validation results rather than on training accuracy alone. Particular attention was given to both the mean test performance and the consistency of the results across folds. This distinction is important for the present dataset because highly flexible models can reproduce a small training set closely without providing a corresponding improvement for withheld configurations.

Following the comparative evaluation, the stability of the reference Ridge model was assessed on the fixed nine-case hold-out set using non-parametric bootstrap refitting. A total of 1000 bootstrap datasets were generated from the original 36-case training set. In each bootstrap repetition, 36 observations were sampled with replacement from the training data, after which the complete pre-processing and Ridge-regression pipeline was refitted to the resampled dataset. The refitted model was subsequently applied to the same nine hold-out configurations. For each hold-out case, the mean prediction, standard deviation, and coefficient of variation were calculated from the 1000 bootstrap predictions. The coefficient of variation was calculated as Equation (47):

(47) $$\mathrm{CV}=100\;\frac{S_{\mathrm{boot}}\;}{{\bar{y}}_{\mathrm{boot}}}$$

where *S_boot_* and ${\bar{y}}_{\mathrm{boot}}$ are the standard deviation and mean of the bootstrap predictions, respectively. The 95% bootstrap intervals reported for the fitted Ridge coefficients were determined from the 2.5th and 97.5th percentiles of the corresponding bootstrap distributions. The bootstrap analysis therefore quantifies the sensitivity of the fitted model to resampling of the available training observations rather than uncertainty arising from structural modelling, loading, material properties, or the stochastic optimization process.

## 3. Results and Discussion

### 3.1. Optimization-Based Dataset Generation and Algorithm Behaviour

The optimization analyses were conducted for the full factorial combination of three storey numbers (17, 24, and 30), three bracing types (V, X, and K), and five brace-placement layouts, resulting in 45 structural configurations. Each configuration was analysed once using HBA, AO, GWO, and PSO, giving 180 optimization runs in total. For a given configuration, the lowest feasible structural weight obtained from the four algorithm-specific searches was retained as the best-observed feasible weight and subsequently used as the response variable in the machine-learning dataset.

The structural weights obtained for the 17-, 24-, and 30-storey frames are reported in Table 3, Table 4 and Table 5, respectively. All values correspond to feasible designs satisfying the constraints described in Section 2.1.3. The same population size, maximum iteration limit, penalty formulation, and structural analysis procedure were used for all four algorithms, as described in Section 2.2.5 and summarized in Table 2. The brace-placement notation follows the definitions introduced in Section 2.1.1; for example, 1-1 denotes one braced storey followed by one unbraced storey, whereas 2-1 denotes two consecutive braced storeys followed by one unbraced storey.

**Figure 3:**
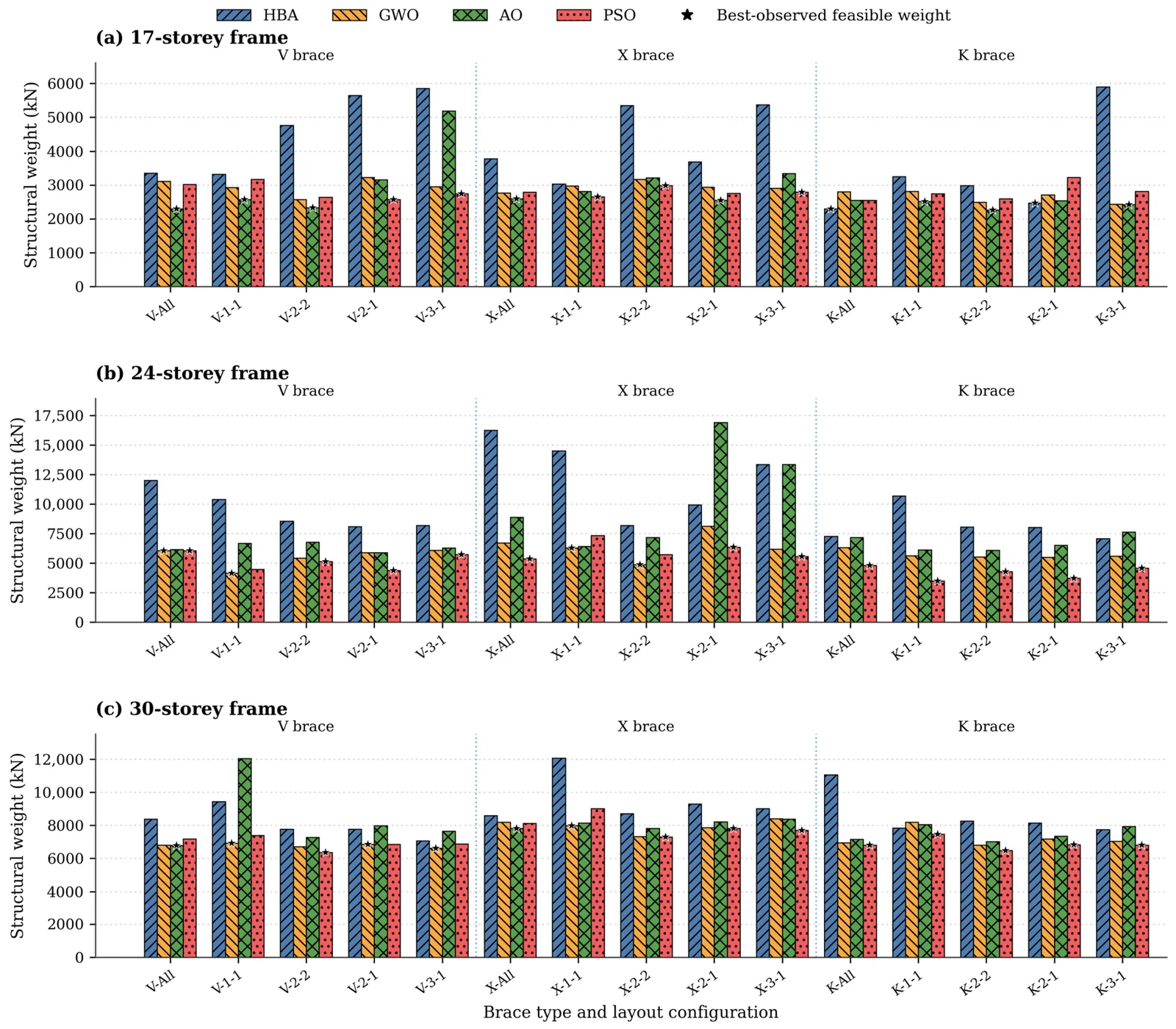
Comparison of the structural weights obtained using the four metaheuristic algorithms for different brace-type and layout configurations of the 17-, 24-, and 30-storey frames.

The numerical results in Table 3, Table 4 and Table 5 reveal a marked dependence of the algorithm-specific solutions on the structural configuration. Figure 3 provides a direct comparison of the weights returned by HBA, GWO, AO, and PSO for all 45 cases. For the 17-storey frames, AO produced the lowest observed weight in eight of the 15 configurations, PSO in five, and HBA in two. For the 24-storey frames, PSO produced the lowest value in 11 configurations and shared the lowest value with GWO for the V-All configuration, while GWO alone produced the lowest value in the remaining three. For the 30-storey frames, PSO produced the lowest value in nine cases, GWO in four, and AO in two. Across the complete set of 45 configurations, PSO therefore provided the lowest observed weight in 25 cases and shared one additional minimum with GWO; AO provided the lowest value in ten cases, GWO in seven cases plus the shared minimum, and HBA in two cases.

The frequency with which an algorithm produced the lowest observed value describes the present 180 runs; a rank-based comparison over the 45 configurations is given below. Nevertheless, Figure 3 and Table 3, Table 4 and Table 5 show that reliance on a single algorithm would have changed the retained design weight for many configurations. PSO produced the lowest observed weight most frequently, particularly for the 24- and 30-storey frames, whereas AO was more competitive for the 17-storey cases. HBA returned comparatively high weights in many configurations, most notably for the 24-storey frames. These differences indicate that the search trajectory and convergence behaviour of the algorithms interact with the discrete structural design space. They also support the decision to retain the lowest feasible result from the four searches rather than construct the subsequent dataset from one arbitrarily selected metaheuristic.

Because each algorithm was executed once for each configuration, the four results available for a configuration are four independent stochastic searches of the same design problem rather than repeated runs of one algorithm. The 45 configurations therefore form a matched-block design, and the algorithms can be compared over the blocks without additional runs. Table 6 summarizes the weights returned by each algorithm over the 45 configurations. The spread among the four searches of a configuration is appreciable: the coefficient of variation averages 22.3% and ranges from 3.8% to 52.2%, and the mean difference between the highest and lowest of the four values is 67.3% of the lowest. This dispersion is the reason the lowest of the four searches, rather than the result of any single algorithm, was retained as the target of the machine-learning stage.

A rank-based comparison over the 45 blocks was carried out following the protocol recommended for metaheuristic comparisons [74]. The Friedman test [75] rejects the hypothesis of equal performance, with a chi-square statistic of 60.90 on three degrees of freedom and *p* = 3.8 × 10^−13^; the corresponding Kendall coefficient of concordance is 0.451. The mean ranks are 1.68 for PSO, 2.10 for GWO, 2.53 for AO and 3.69 for HBA. Pairwise Wilcoxon signed-rank tests [76] with the Holm correction [77] give an adjusted *p*-value below 0.05 for all six pairs, the largest being 0.017 for GWO against PSO. Because the weights differ in scale between the height groups, the pairwise tests were repeated on the weights normalized by the lowest value of each configuration, and all six comparisons remain significant, the largest adjusted *p*-value being 0.018. The differences between the algorithms are therefore systematic rather than an artefact of the particular random initializations. Applied within each height group, the Friedman test also rejects equal performance, with *p* = 4.5 × 10^−4^ at 17 storeys, 3.2 × 10^−8^ at 24 storeys and 4.5 × 10^−5^ at 30 storeys, although the ordering is not the same in the three groups: AO attains the best mean rank at 17 storeys and PSO at 24 and 30 storeys. As an independent check, 25 lowest values out of 45 would occur with probability 1.2 × 10^−5^ if the four algorithms were equally likely to produce the lowest weight.

These results establish an ordering on the present design problems under the adopted settings and computational budget. They do not measure the run-to-run variability of a single algorithm, which would require repeated runs with different random seeds, and they should not be read as a general ranking of the four algorithms.

All 45 retained designs satisfy Equations (2)–(10), so V = 0 and Equation (42) reduces to the structural weight for every value used as a machine-learning target. The weights reported in Table 3, Table 4 and Table 5 are therefore structural weights rather than penalized fitness values, and they do not depend on the penalty exponent. The exponent affects the trajectory of the search rather than the evaluation of the designs that were retained.

When the lowest feasible result in each row of Table 3, Table 4 and Table 5 is considered, the mean best-observed feasible weight increases from 2537 kN for the 17-storey frames to 4983 kN for the 24-storey frames and 7103 kN for the 30-storey frames. The corresponding increase is approximately 96% between the 17- and 24-storey groups and 43% between the 24- and 30-storey groups, giving an overall increase of approximately 180% between 17 and 30 storeys. The ranges are 2264–2987 kN, 3503–6341 kN, and 6366–7970 kN for the 17-, 24-, and 30-storey models, respectively. Thus, storey-number group is the dominant discriminator of structural weight within the investigated dataset.

The cross-height trend should, however, be interpreted within the loading assumptions adopted in Section 2.1.2. Wind loading was included for the 24- and 30-storey models but not for the 17-storey models. Consequently, the difference between the 17-storey group and the two taller groups reflects both the increase in building height and the change in the applied loading regime; it cannot be attributed exclusively to height. The weight trends reported here apply to the structural-analysis and design assumptions adopted in this study.

Bracing type produces a smaller but still substantial variation in the retained structural weight. Averaged over the five layouts, X-bracing gives mean best-observed feasible weights of 2716, 5690, and 7721 kN for the 17-, 24-, and 30-storey frames, respectively, and is the heaviest of the three brace types at every investigated height. K-bracing gives the lowest mean weight for the 17- and 24-storey groups, at 2393 and 4175 kN, respectively. For the 30-storey frames, the mean weights of V- and K-bracing are 6713 and 6875 kN, respectively, corresponding to a difference of only approximately 2.4%. The relative difference between the heaviest and lightest mean bracing types is approximately 13% at 17 storeys, 30% at 24 storeys, and 14% at 30 storeys.

The greater weight obtained for X-bracing does not imply inferior lateral performance. X-bracing is commonly associated with high lateral stiffness and reduced drift demand [49]. The present results instead show that stiffness alone is not a sufficient indicator of minimum-weight efficiency. In a constrained weight optimization problem, brace geometry also changes the quantity of diagonal steel, axial-force paths, and the forces transferred to beams and columns; these effects can offset the weight benefit associated with greater lateral stiffness. The present comparison therefore concerns total optimized frame weight under the adopted constraints rather than lateral stiffness considered in isolation.

The lightest individual configuration also changes with building height. For the 17-storey frames, the lowest observed feasible weight is 2264.085 kN for K-bracing with the 2-2 layout, obtained by AO. For the 24-storey frames, the corresponding minimum is 3502.945 kN for K-bracing with the 1-1 layout, obtained by PSO. For the 30-storey frames, the lowest value is 6366.247 kN for V-bracing with the 2-2 layout, also obtained by PSO. The lowest K-braced value at 30 storeys is 6470.841 kN, only 104.594 kN above the V-braced minimum. These results confirm that a brace type identified as weight-efficient at one height should not automatically be assumed to remain the preferred alternative for a taller frame.

Brace-placement layout has the smallest average influence of the three configuration variables, but its effect is not negligible. Averaged over the three bracing types, the difference between the lightest and heaviest layouts is approximately 256 kN for the 17-storey frames, 764 kN for the 24-storey frames, and 744 kN for the 30-storey frames. These differences correspond to approximately 10–15% of the mean weight of the respective height groups. The layout producing the lowest average weight also changes with height: All is the lightest average layout for the 17-storey group, 1-1 for the 24-storey group, and 2-2 for the 30-storey group.

This variation is consistent with the structural role of vertical brace distribution. Changing the sequence of braced and unbraced storeys alters the stiffness profile and the transfer of axial and lateral forces along the height, which in turn changes the member sections required to satisfy the strength and drift constraints. A layout that reduces material demand in one height group can therefore redistribute demand unfavourably in another. The results do not support selection of a single brace-placement pattern independently of building height and bracing type.

Taken together, Table 3, Table 4 and Table 5 and Figure 3 show that storey-number group governs the overall scale of structural weight in the present dataset, while bracing type provides the larger of the two configuration-dependent design effects and brace placement produces a smaller but non-uniform contribution. The 45 best-observed feasible weights obtained by retaining the lowest feasible result from HBA, AO, GWO, and PSO, together with their associated storey number, bracing type, and brace-placement layout, constitute the dataset used for the machine-learning analyses presented in Section 3.2. These comparisons, and the regression models fitted to them, refer to one fixed parameterization of the design problem, because the same member grouping was applied to all 45 configurations. A finer grouping would be expected to lower all 45 targets; whether it would change their relative ordering is not established by the present results.

### 3.2. Performance Evaluation and Design-Level Implications of the Machine-Learning Models

The predictive performance of the six regression models was evaluated using the best-observed feasible weights obtained from the optimization analyses described in Section 3.1. The comparison was conducted in terms of the agreement between observed and predicted structural weights, five-fold cross-validation performance, residual behaviour, and the stability of predictions under bootstrap refitting. Particular attention was given to whether the additional flexibility of the ensemble and distance-based models improved prediction relative to the simpler Ridge model, and to the extent to which the fitted relationships could be interpreted in terms of the governing structural configuration parameters.

The hyperparameters used for each regression algorithm were held fixed throughout the evaluation procedure and are summarized in Table 7. The same pre-processing pipeline described in Section 2.3 was applied within each model evaluation so that categorical encoding and standardization were performed consistently.

**Figure 4:**
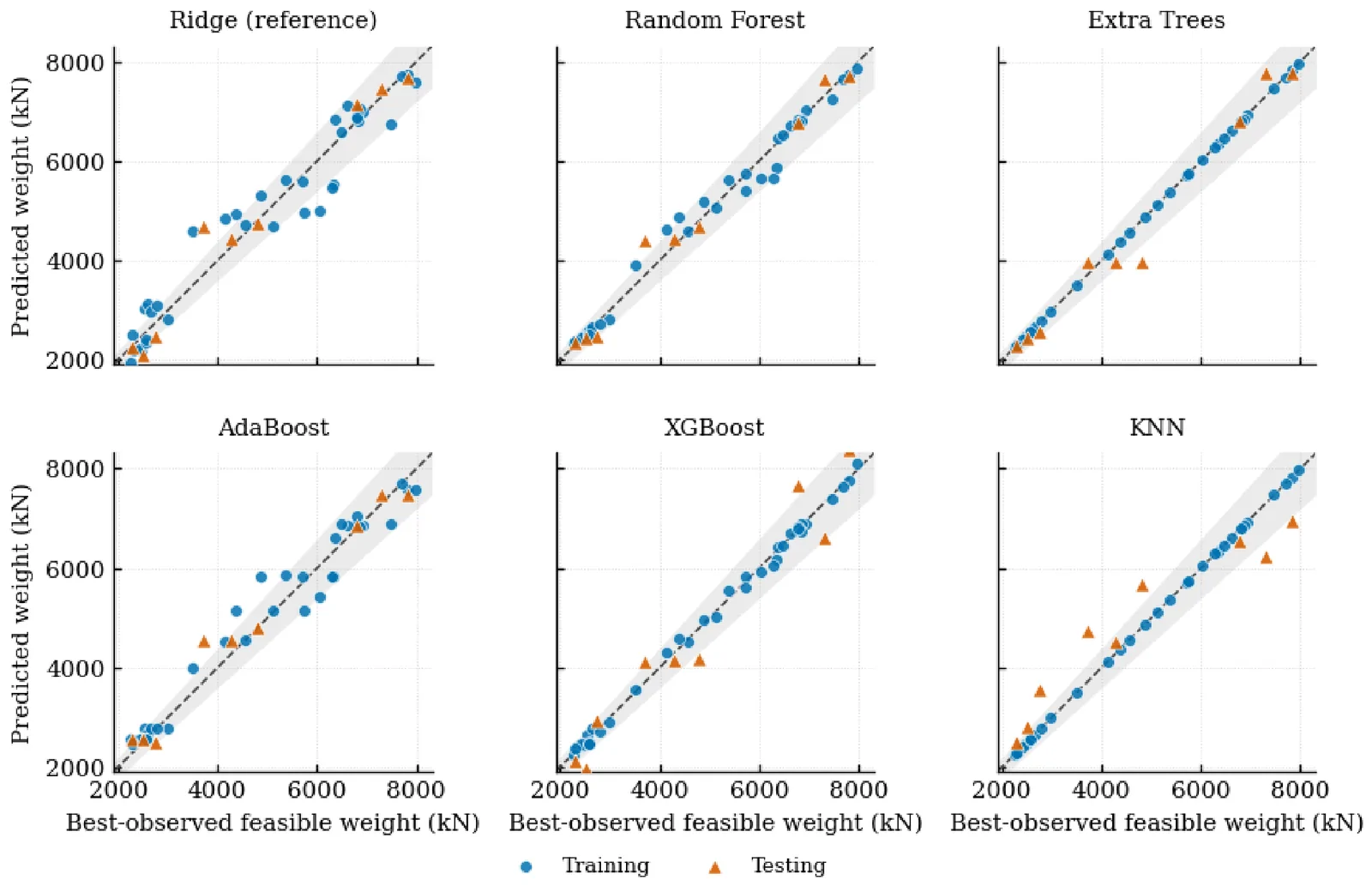
Comparison of observed and predicted best-observed feasible structural weights obtained using the six regression models.

Figure 4 compares the best-observed feasible weights with the corresponding predictions obtained from the six regression models for the fixed 80/20 train–test partition described in Section 2.3. The diagonal line represents exact agreement between prediction and observation, while the shaded region provides a common visual reference around this line.

The models exhibit markedly different behaviour between the training and hold-out cases. Random Forest, Extra Trees, and XGBoost reproduce the training observations closely, with most training points lying near the diagonal. Their hold-out predictions show greater dispersion, particularly in the intermediate weight range. Extra Trees achieves an exact fit to the training observations, whereas XGBoost also approaches an exact training fit. The corresponding deterioration on held-out data indicates that the additional model flexibility does not translate directly into improved prediction for configurations excluded from fitting.

KNN also reproduces the training observations exactly. This behaviour should not by itself be interpreted as evidence of overfitting because, with distance-based weighting, a training observation is located at zero distance from itself and can consequently dominate its own fitted prediction. Its hold-out behaviour is more informative: several test cases depart appreciably from the diagonal, particularly towards the upper part of the weight range. AdaBoost produces a more discrete prediction pattern, which reflects the piecewise nature of the decision-tree learners forming the boosted ensemble.

Ridge displays a different train–test pattern. Its training predictions retain visible residual scatter, but the hold-out points occupy broadly the same prediction region and show no comparable collapse in predictive performance. This behaviour is consistent with the cross-validation results presented in Table 8 and supports the use of Ridge as the reference model for subsequent interpretation. Importantly, the hold-out configurations are combinations of factor levels already represented in the dataset; they should therefore be regarded as held-out configurations within the investigated design domain rather than genuinely new structural systems.

The complete five-fold cross-validation results are reported in Table 8. The metrics calculated using Equations (43)–(46) are presented separately for the training and test partitions of each fold.

Table 8 shows that Ridge yields the highest mean test *R*^2^, at 0.914, closely followed by Random Forest at 0.913. Random Forest, however, gives the lowest mean test RMSE and MAE, at 511.7 and 343.1 kN, respectively, compared with 523.0 and 398.0 kN for Ridge. The difference of only 0.001 in mean test *R*^2^ does not provide a basis for claiming that one of these two models is unequivocally more accurate. Rather, the two provide comparable mean predictive performance according to *R*^2^, with Random Forest giving somewhat smaller absolute errors and Ridge providing a simpler, directly interpretable relationship.

The fold-to-fold behaviour provides an additional distinction. Ridge test *R*^2^ ranges from 0.856 to 0.963, whereas Random Forest ranges from 0.832 to 0.980. The standard deviation of the five test *R*^2^ values is approximately 0.049 for Ridge and 0.060 for Random Forest. Ridge was therefore retained as the reference model because it combined the highest mean test *R*^2^, relatively low fold-to-fold variability, and a form that can be expressed directly in terms of the three structural configuration variables. The two models are compared directly below.

The more flexible models exhibit a larger separation between training and held-out performance. Extra Trees fits every training fold exactly but yields a mean test *R*^2^ of 0.853. XGBoost reaches a mean training *R*^2^ of 0.998 but decreases to 0.832 on the test folds. KNN has a training *R*^2^ of 1.000 and a mean test *R*^2^ of 0.737. As noted above, the exact KNN training fit is partly inherent to distance-weighted nearest-neighbour prediction and should not be used alone as evidence of overfitting. Its comparatively high test RMSE and MAE nevertheless show that local similarity in the encoded feature space is less effective for predicting the held-out configurations in this dataset.

The largest deterioration in predictive performance occurs in fold 2 for all six models. Ridge remains the least affected in relative terms, with a test *R*^2^ of 0.856, compared with 0.832 for Random Forest, 0.742 for AdaBoost, 0.706 for Extra Trees, 0.642 for XGBoost, and 0.555 for KNN. The common reduction across models indicates that the composition of this fold contains configurations that are intrinsically more difficult to predict from the remaining cases rather than a weakness unique to one learning algorithm. Given the limited dataset of 45 observations, variation between folds is therefore an important component of model assessment.

Because the values reported in Table 8 are obtained from a single five-fold partition, the cross-validation was repeated 1000 times with different fold assignments. The resulting distribution is given in Table 9. For Ridge the mean test *R*^2^ is 0.904, with a 95% interval of 0.859 to 0.927, and the value of 0.914 reported in Table 8 exceeds approximately 70% of the repetitions. Ridge also shows the smallest dispersion of the six models, with a standard deviation of 0.022 against 0.033 for Random Forest, 0.050 for Extra Trees, and 0.058 for KNN.

A paired comparison between the two leading models was also carried out. Each of the 45 configurations was predicted from the remaining 44, and Ridge was compared with Random Forest by applying the Wilcoxon signed-rank test [76] to the 45 matched configurations, following the protocol adopted in Section 3.1 [74]. The test gives *p* = 0.26 for the absolute error and *p* = 0.27 for the squared error, so the difference in accuracy between the two models is not statistically significant. The two models differ in the shape of the error distribution rather than in its size: Random Forest gives the smaller mean absolute error, 368 against 410 kN, whereas Ridge gives the smaller root-mean-square error, 533 against 548 kN, and the smaller largest error, 1321 against 1491 kN. Ridge was retained as the reference model because its accuracy is statistically indistinguishable from that of the ensemble models, its dispersion over the repeated partitions is the smallest in Table 9, its largest errors are smaller, and its fitted relationship can be written directly in terms of the three configuration variables.

Two further checks were carried out on the adequacy of the dataset. Permuting the target values 1000 times and re-evaluating the Ridge pipeline gives a null distribution with a mean *R*^2^ of −0.506 and a maximum of 0.145, so the observed value lies well outside the range attainable by chance (*p* = 0.001). Refitting the model to random subsets gives a median cross-validated *R*^2^ that rises from 0.525 for 15 configurations to 0.859 for 25, and then changes slowly, reaching 0.909 for the complete set of 45. Subsets that omit a factor level from a fold produce large negative values, and the proportion of such subsets falls from 0.33 at 15 configurations to zero at 35 and above. The dataset therefore lies beyond the steep part of the learning curve, and the complete factorial arrangement ensures that every factor level is represented in each fold.

The separation between training and test performance in Table 8 supports the same conclusion for the reference model. The difference between mean training and mean test *R*^2^ is 0.036 for Ridge, compared with 0.076 for Random Forest, 0.085 for AdaBoost, 0.147 for Extra Trees, 0.166 for XGBoost, and 0.263 for KNN. The optimism associated with a small dataset therefore affects the more flexible models considerably more than the additive model retained here. These checks concern predictive performance within the investigated design domain; generalization to configurations outside that domain is a separate question and is considered below.

**Figure 5:**
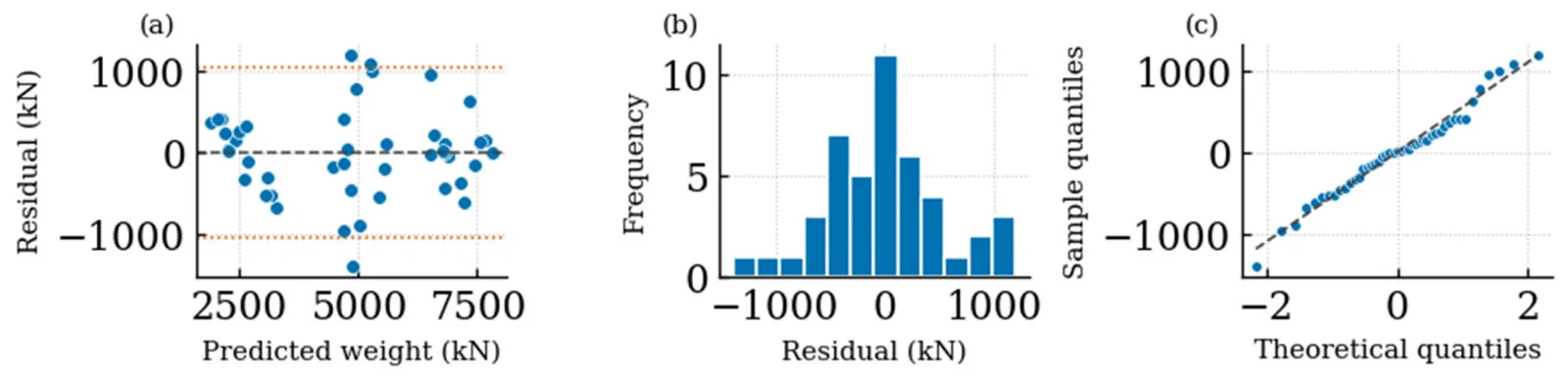
Residual diagnostics of the reference Ridge model obtained from five-fold cross-validation: (**a**) residuals versus predicted weight, with the dashed line at zero residual and the dotted lines at the ±1.96-standard-deviation limits; (**b**) residual distribution; and (**c**) normal quantile–quantile plot, with the dashed line showing the normal reference line.

The residual diagnostics obtained from the cross-validated Ridge predictions are presented in Figure 5. The residuals are considered as the differences between the observed and predicted structural weights.

Figure 5a shows that the predicted values form three principal clusters corresponding to the 17-, 24-, and 30-storey groups. This separation is consistent with the large between-height differences identified in Section 3.1. The residuals occur on both sides of zero within each cluster, and no pronounced monotonic trend with predicted weight is evident. The plotted ±1.96-standard-deviation limits correspond to approximately ±1100 kN and are used here as descriptive reference limits rather than as formal confidence bounds. Most observations fall within this region, although a small number of cases show larger deviations.

The residual distribution in Figure 5b is concentrated around zero, with approximately two-thirds of the cases lying within ±400 kN. The negative tail extends to approximately −1400 kN and is somewhat longer than the positive tail, which reaches approximately +1200 kN. Figure 5c shows that the central portion of the residual distribution follows the normal-reference line reasonably closely, while a departure is visible towards the lower tail. The diagnostic plots therefore do not indicate a strong prediction trend, but neither do they support an assumption of perfectly normally distributed errors. The relatively small sample and the clustering associated with the three investigated storey levels should also be considered when interpreting these residual diagnostics.

**Figure 6:**
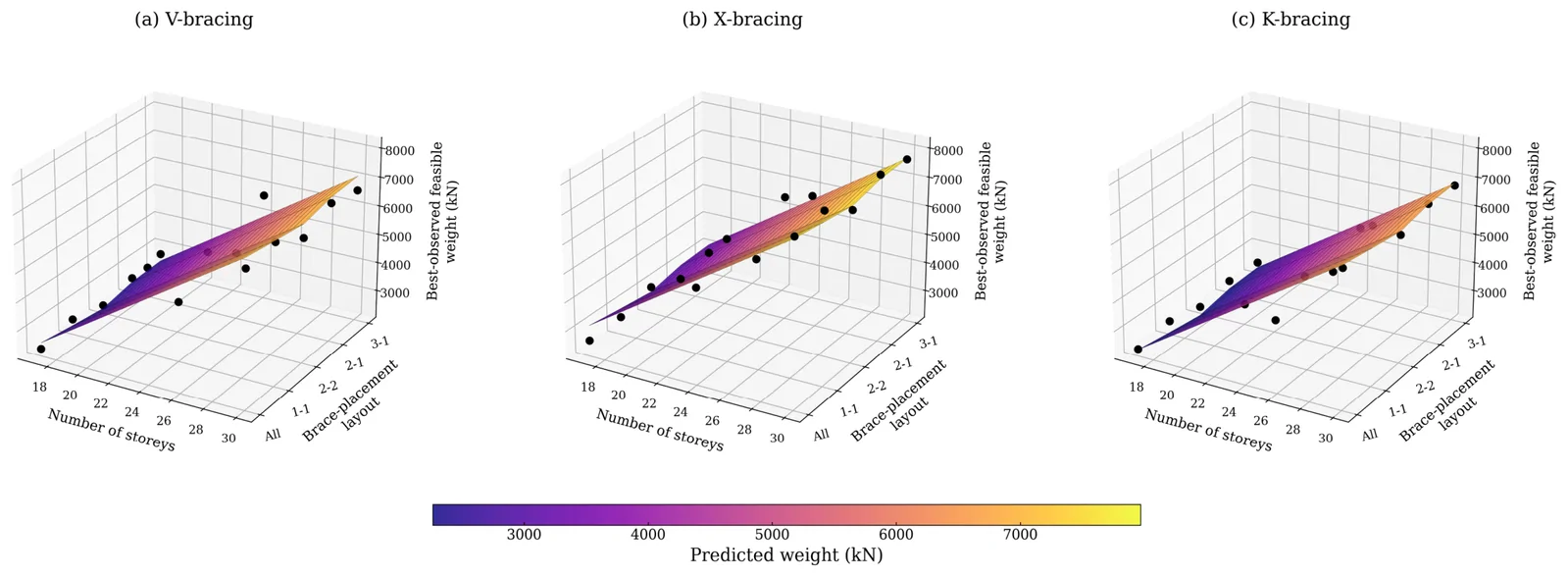
Ridge response surfaces relating predicted best-observed feasible weight to storey number and brace-placement layout for the three bracing types.

To examine the relationship learned by the reference model, Figure 6 presents the Ridge response surfaces as functions of storey number and brace-placement layout separately for V-, X-, and K-bracing.

The observed optimization results are superimposed on the surfaces in Figure 6. All three surfaces rise markedly as the number of storeys increases, whereas the vertical shifts associated with bracing type and brace-placement layout are smaller. Because Ridge was fitted as an additive model without interaction terms, the response surfaces are approximately planar and the bracing and layout effects remain constant with storey number in the fitted equation.

The standardized coefficient associated with storey number is approximately 1861 kN, meaning that an increase of one standard deviation in the storey-number variable, approximately 5.31 storeys for the present dataset, increases the fitted weight by about 1860 kN. The corresponding bootstrap refits produced an interval of approximately 1700–1910 kN. This strong model coefficient agrees with the clear separation among the three height groups observed in Section 3.1. It should not, however, be interpreted as a causal effect of building height, because the three groups differ in several respects at once. The 17-storey models were analysed without wind loading, whereas wind was included for the 24- and 30-storey models; the storey height is 3.0 m in the 17- and 24-storey models and 3.66 m in the 30-storey model; and the three plans differ in shape and in area. The fitted storey-number coefficient therefore carries the combined effect of the number of storeys, the storey height, the plan geometry and the loading regime.

The magnitude of this combined effect can be separated from the variation that occurs within a height group. The grouping accounts for 90.8% of the total sum of squares of the best-observed feasible weight, leaving 9.2% for bracing type and brace-placement layout. The between-group part does not scale with the number of storeys: from 17 to 24 storeys the storey count rises by 41% while the mean weight rises by 96%, and from 24 to 30 storeys the corresponding figures are 25% and 43%. The plan areas move in the opposite direction over part of this range, with the typical lower-zone floor covering 180 m^2^ in the 17-storey model, 405 m^2^ in the 24-storey model and 214 m^2^ in the 30-storey model. Comparisons between the groups therefore describe three different structural systems rather than the same system at three heights.

Comparisons between bracing types and between brace-placement layouts are made within a height group, where the loading, the storey height and the plan are identical, and are not affected by the differences between the groups. Within the groups, the spread of the mean weight between bracing types is 12.7%, 30.4% and 14.2% of the group mean at 17, 24 and 30 storeys, and the spread between brace-placement layouts is 10.1%, 15.3% and 10.5%, with X-bracing giving the heaviest mean weight in all three groups. A sensitivity analysis using a consistent wind-loading strategy at all three heights would require the optimization to be repeated for the full dataset and is identified as a direction for further work.

The mean bracing effects are smaller than the storey-number coefficient. Relative to the centred bracing effect, X-bracing increases the fitted weight by approximately 498 kN, K-bracing decreases it by approximately 391 kN, and V-bracing has a smaller negative offset of approximately 107 kN. The bootstrap intervals reported for X- and K-bracing are approximately 290–670 kN and −570 to −190 kN, respectively. The layout coefficients are smaller still, and the corresponding bootstrap intervals include zero for all five layout categories. These results are consistent with the descriptive comparison in Section 3.1, in which bracing type produced a larger weight variation than brace-placement layout.

The additive structure of Ridge nevertheless imposes an important restriction on this interpretation. The descriptive results in Table 3, Table 4 and Table 5 show that the relative performance of V-, K-, and X-bracing changes with building height, particularly for V- and K-bracing at 30 storeys. A model containing only additive main effects cannot reproduce such height-dependent changes in the bracing coefficients. The Ridge terms should therefore be interpreted as average effects over the investigated 45 configurations rather than as fixed structural effects that necessarily remain unchanged at every height.

For direct preliminary estimation, the Ridge model can be back-transformed from the standardized storey-number scale to the original number of storeys. The resulting fitted relationship is given by Equation (48):

*W*_pred_ = 350.369*n*_s_ − 3417.537 + *c*_b_ + *c*_l_ (48)

where *W*_pred_ is the predicted best-observed feasible structural weight in kN and *n*_s_ is the number of storeys. The bracing coefficient *c*_b_ is −107.231 kN for V-bracing, −390.873 kN for K-bracing, and +498.104 kN for X-bracing. The layout coefficient *c*_l_ is +104.138 kN for All, +19.887 kN for 1-1, −37.134 kN for 2-1, −205.220 kN for 2-2, and +118.329 kN for 3-1. With these coefficients reported to three decimal places, Equation (48) reproduces the predictions of the fitted Ridge model to within approximately 0.01 kN.

Equation (48) is applicable only to the design domain represented in the present dataset: storey numbers between 17 and 30 and the V-, X-, and K-bracing systems with the five investigated layouts. Because only three discrete storey levels were used for model development, application of Equation (48) to intermediate storey numbers constitutes interpolation of the fitted linear trend rather than validation at those heights. Its use outside the 17–30-storey range, for different bracing systems, or under loading and modelling assumptions different from those adopted here would constitute extrapolation and is not supported by the present results. Under seismic design conditions this restriction is particularly clear: as described in Section 2.1.2, the governing constraints and the admissible brace arrangements both change, so the coefficients of Equation (48) would not carry over. The procedure that produced them is not tied to a particular loading definition and can be repeated for a dataset generated under seismic provisions. The effect of a bracing type or a layout that is absent from the training set is quantified below.

**Figure 7:**
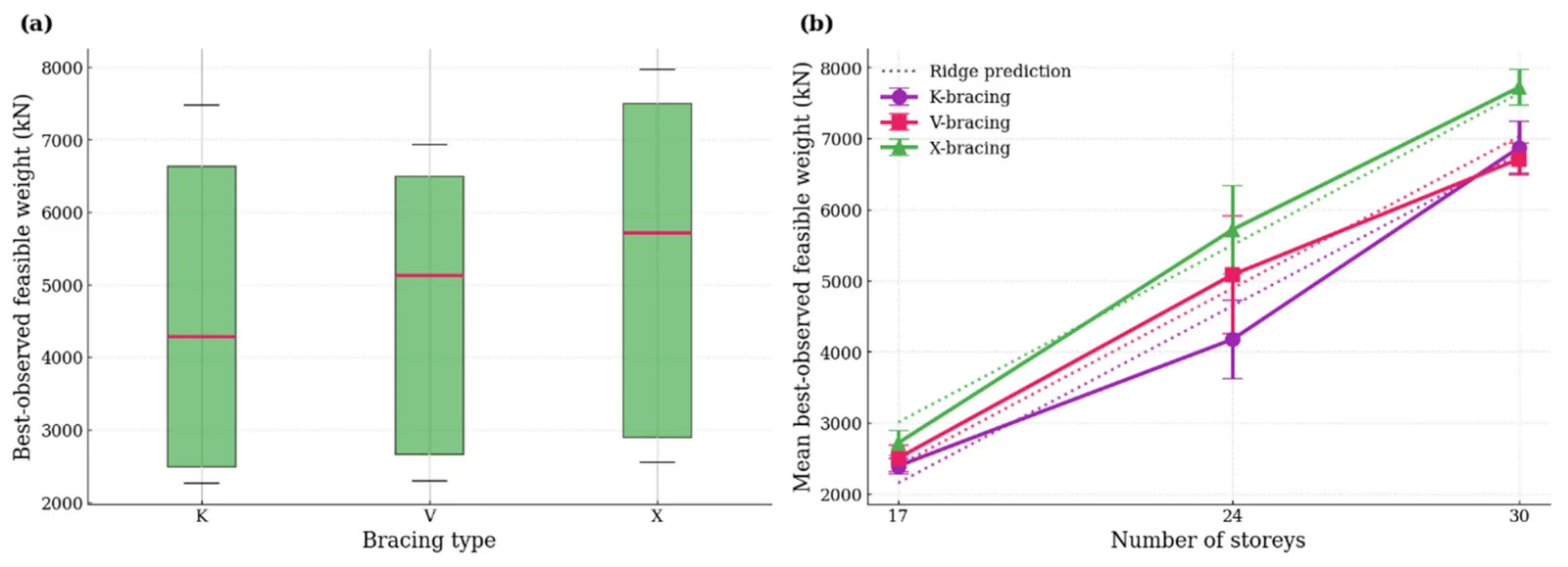
Effect of bracing type and storey number on the best-observed feasible structural weight: (**a**) weight distribution by bracing type and (**b**) mean weight variation with storey number, where the solid lines show the observed means and the dotted lines of the same colour show the corresponding Ridge predictions.

The combined effects of bracing type and storey number are examined further in Figure 7.

The distributions in Figure 7a overlap substantially. K-bracing has the lowest overall median weight, at approximately 4300 kN, V-bracing has a median of approximately 5100 kN, and X-bracing has the highest median, at approximately 5700 kN, together with the broadest overall range. The overlap demonstrates that bracing type alone does not determine the resulting structural weight; its effect depends on the associated height and brace-placement configuration.

Figure 7b compares the mean observed weight for each bracing type at the three storey levels with the corresponding Ridge predictions averaged over the five layouts. At 17 storeys, the observed means are approximately 2400–2700 kN and lie close to the fitted trends. At 24 storeys, the observed K-bracing mean, approximately 4175 kN, lies below the additive Ridge prediction to a greater extent than the corresponding V- and X-bracing means. At 30 storeys, the observed mean weights of V- and K-bracing converge to approximately 6713 and 6875 kN, respectively. The empirical effect of brace type is therefore not identical at all heights, while the overall Ridge model retains high cross-validated predictive accuracy.

**Figure 8:**
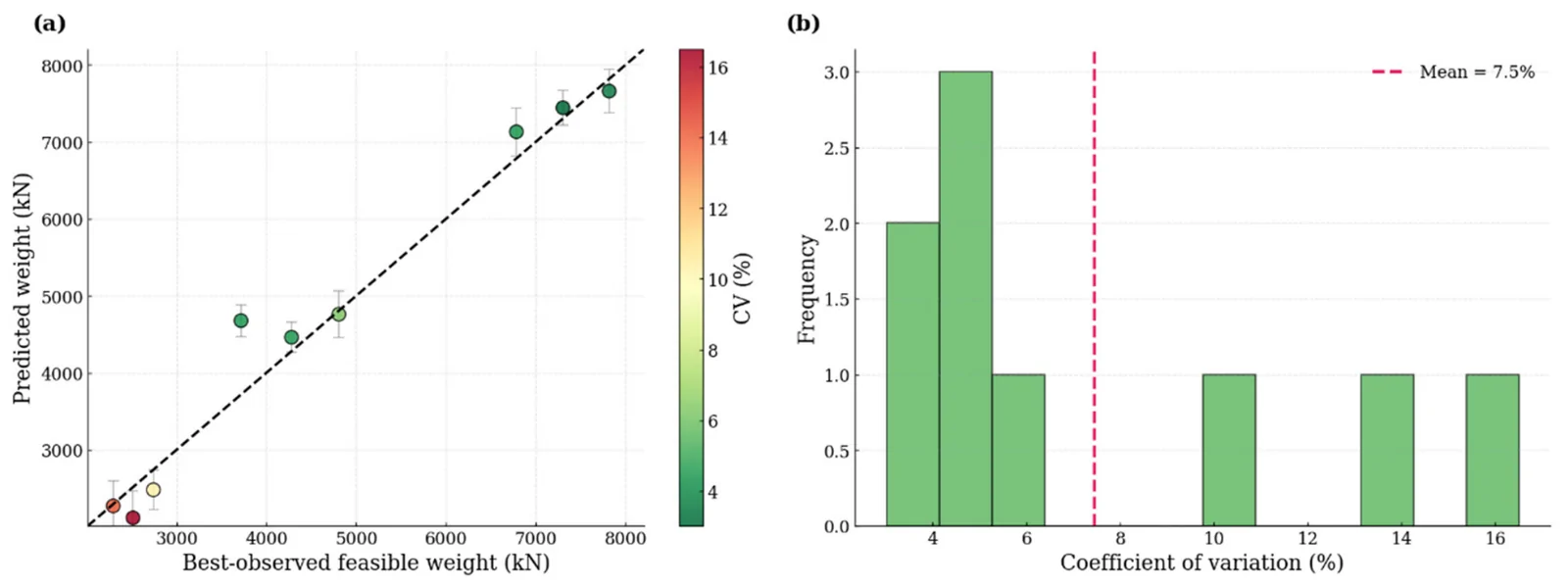
Bootstrap-based variability of the Ridge predictions for the hold-out test set: (**a**) predicted versus best-observed feasible weight with one-standard-deviation bootstrap dispersion, where the marker colour indicates the coefficient of variation and the dashed line marks equality between predicted and observed values, and (**b**) distribution of the coefficient of variation across the hold-out cases.

Finally, Figure 8 examines the sensitivity of the Ridge predictions to bootstrap refitting using the fixed nine-case hold-out set.

In Figure 8a, the error bars represent one standard deviation of the predictions obtained across the bootstrap-refitted Ridge models, while the coefficient of variation (CV) expresses this dispersion relative to the corresponding mean prediction. Seven of the nine hold-out configurations have CV values below 6%. The two lowest-weight hold-out cases, located near 2500–2600 kN, exhibit substantially larger variability, with CV values of approximately 10% and 16–17%. Figure 8b shows the same pattern more directly, with a mean CV of approximately 7.5% across the nine hold-out cases.

The bootstrap results indicate that the fitted Ridge relationship is relatively insensitive to resampling for most of the hold-out configurations but becomes less stable near the lower boundary of the sampled weight range. The bootstrap dispersion should be interpreted as sensitivity of the fitted model to the available sample rather than as a complete prediction-uncertainty interval, because it does not incorporate uncertainty in structural modelling, loading assumptions, material properties, or the stochastic optimization targets themselves. It is also not uniformly smaller than the differences among competing structural configurations; for example, the mean V- and K-bracing weights at 30 storeys differ by only about 2.4%. Consequently, configurations with small predicted weight differences should not be ranked solely on the basis of the surrogate prediction.

The response variable is the lowest of four algorithm-specific searches and therefore depends on the set of algorithms used. That dependence was examined by removing one algorithm at a time and repeating the analysis with the lowest of the remaining three. Table 10 reports the outcome. Removing HBA, GWO or AO leaves the target unchanged in 43, 38 and 35 of the 45 configurations and raises it on average by 0.3%, 0.9% and 2.1%; removing PSO, which produced the lowest value most often, leaves 20 configurations unchanged and raises the target by 8.5% on average. The rank correlation with the full target remains between 0.965 and 0.998, and the mean test *R*^2^ lies between 0.834 and 0.911 against 0.904 for the full target. The fitted coefficients keep the same structure: the storey-number coefficient stays between 1809 and 1912 kN, X-bracing retains the largest positive bracing coefficient, and the remaining bracing coefficients stay negative.

Replacing the lowest value with a summary of the four searches was also examined. Training on their mean gives a mean test *R*^2^ of 0.641 and training on their median gives 0.731, against 0.904 for the lowest value. The four searches differ mainly in how far each fell short of convergence, and the lowest of them is the value closest to the solution of the design problem defined by Equations (1)–(10), so it carries the clearest structural signal; a mean taken over algorithms of unequal quality combines that solution with the shortfall of the weaker searches. A mean over four different algorithms is in any case not an estimate of the run-to-run variability of a single algorithm. Predicting a distribution of outcomes rather than a single value would require repeated runs of each algorithm with different random seeds.

The sources of uncertainty in a predicted weight can be separated into three groups. The first is the sample of 45 configurations from which the relationship is fitted. Refitting the model to bootstrap resamples of the remaining 44 configurations and predicting each configuration in turn gives a mean standard deviation of 224 kN, which is the bootstrap dispersion of Figure 8 evaluated over all 45 configurations rather than over the nine hold-out cases. The second is the stochastic optimization that defines the target: replacing the four searches by the best of any three changes the target with a mean standard deviation of 248 kN and the fitted predictions by 139 kN on average, rising to 393 kN when PSO, the search that produced the lowest value most often, is removed. The third group comprises the structural model, the loading assumptions and the material properties. The material properties are the same for all 45 configurations, and within a height group the structural model and the loading are identical as well, so this group displaces the configurations of a group together rather than altering the comparison between them; between height groups these assumptions differ, which is why the storey-number coefficient is not interpreted as a height effect. This group cannot be estimated from the present dataset, and its scope is set by the assumptions stated in Section 2.

No single source dominates the predicted weight of an individual configuration: the sampling of the dataset and the variability of the target contribute in similar measure, 224 and 248 kN, and both are smaller than the leave-one-out mean absolute error of 410 kN. For the comparison of configurations within a height group, which is the use identified below, the third group does not contribute because it is common to them. Of the three, the stochastic optimization is the one that a further study could reduce directly, by repeating each optimization with several random seeds, which is identified as a direction for further work.

The 45 configurations form a complete factorial arrangement of three storey numbers, three bracing types and five brace-placement layouts, so a randomly drawn test case combines levels of the three variables that are all represented in the training set. The random partitions used above therefore measure interpolation within the investigated design domain. To examine what happens when a level itself is absent from the training set, the model was refitted three further times with one storey number withheld, three times with one bracing type withheld and five times with one layout withheld, and each withheld group was then predicted. Table 11 reports the result in the units of the objective function, because the coefficient of determination is not informative for a single withheld group: the grouping accounts for 90.8% of the total variation, as reported above, so the variance remaining inside one group is small and the ratio on which the coefficient of determination is based becomes unstable.

A storey number absent from the training set is reproduced accurately at group level. The mean signed error is −15, +12 and −34 kN for the 17-, 24- and 30-storey groups, which is below 0.6% of the corresponding mean weight, and the mean absolute error, between 311 and 631 kN, is of the same order as the 398 kN obtained for the fixed five-fold partition in Table 8. The linear storey term carries this behaviour: a straight line through the mean weights of the 24- and 30-storey groups gives 2510 kN at 17 storeys against the observed group mean of 2537 kN, and a straight line through the 17- and 24-storey group means gives 7080 kN at 30 storeys against the observed 7103 kN. Within the investigated range the storey trend is therefore transferable, although the variation among configurations inside a withheld height is reproduced less closely than inside a height that is represented in the training set.

A bracing type or a layout absent from the training set is a different situation. These variables enter the model through one-hot encoding, so a level that never appears in training has no coefficient and the corresponding configuration is predicted at the average of the levels that were seen. The mean signed error is then the difference between the mean weight of the levels that were retained and that of the level withheld: +590 kN for K-bracing, the lightest type, and −752 kN for X-bracing, the heaviest. The effect is smaller for the layouts, whose mean weights differ less, and stays between −150 and +259 kN. This behaviour is a property of categorical predictors rather than of the fitted model, and it sets the scope of Equation (48): the equation applies to the V-, X- and K-bracing systems and the five layouts investigated here, and a bracing arrangement outside this set requires the dataset to be extended before it can be screened.

The screening use of the fitted model rests on these results rather than on the optimization runs from which the dataset was built. In every partition reported above, the optimization results of the held-out configurations are excluded from the fitting, and the encoding and standardization are carried out inside each training partition; the held-out results enter only as the reference against which the error is measured. What the fitted model is asked to do in practice is not to reproduce a weight that is already known, but to separate configurations that have not been optimized. The quantity that decides whether it can do so is the size of the difference being screened relative to the prediction error obtained when each configuration is predicted from the other 44. Within the 24-storey group the mean weights of the three bracing types span 1515 kN against a mean absolute error of 641 kN, a ratio of 2.4, and within the 30-storey group they span 1009 kN against an error of 261 kN, a ratio of 3.9; within the 17-storey group the span is 323 kN against an error of 327 kN, so bracing alternatives at that height are not separated by the model. A difference smaller than the prediction error, such as the 162 kN between the mean V- and K-bracing weights at 30 storeys noted in Section 3.1, therefore requires the optimization to be carried out.

The computational saving can be stated in terms of the number of optimization runs. Each configuration in the present dataset required four optimization runs, one for each algorithm, each with a population of 30 candidate designs and 50,000 iterations, and the complete dataset required 180 runs. Once the relationship has been fitted, a further configuration within the investigated design domain is screened with one evaluation of Equation (48), without an optimization run. The optimization procedure is then required only for the configurations retained after screening.

The consequence for the selection of a configuration was quantified from the same leave-one-out predictions. Over the 45 configurations the model orders 90.3% of the 990 pairs correctly, with a Kendall rank correlation of 0.81. Within a single height group the figure is 69.5% of 315 pairs, and it follows the size of the difference being resolved: 54% for pairs differing by less than 250 kN and 83% for differences above 1000 kN. Two quantities set this limit. The median difference between two configurations of the same height group is 445 kN, of the same order as the mean absolute error of 410 kN; and the target is the lowest of four stochastic searches whose results for a single configuration have a mean coefficient of variation of 22.3%, as reported in Section 3.1, so part of the ordering within a group follows which search converged best rather than a property of the configuration.

Expressed as a design decision, the model selects the lightest of the 15 configurations of the 17-storey group, the second lightest of the 30-storey group and the fourth lightest of the 24-storey group. The selected weights are 0.0% and 1.6% above the observed minimum in the first two groups and 22.2% in the third, where that minimum is itself 6% below the second lowest value of the group and was returned by one of the four searches while the other three gave between 5631 and 10681 kN. The model therefore identifies the weight level and a competitive configuration within a height group rather than the single lightest one.

The additive form was tested against models containing interaction terms. Ridge models were fitted with a height × bracing interaction, with a height × layout interaction, with both, with a quadratic height term, and with a separate bracing effect for each height group. The interaction terms were formed from the standardized storey variable, and each model was evaluated with the repeated cross-validation of Table 9 and with the leave-one-out predictions used above. Table 12 gives the results. The height × bracing interaction gives the only improvement: the mean test *R*^2^ rises from 0.904 to 0.908 and the leave-one-out mean absolute error falls from 410 to 379 kN, while the effective number of parameters rises from 7.9 to 9.9. Applied to the 45 matched configurations, the paired Wilcoxon signed-rank test gives *p* = 0.11, so the improvement is not established for the present dataset. The height × layout interaction and the quadratic height term both reduce predictive accuracy, the latter significantly.

The convergence of the mean V- and K-bracing weights at 30 storeys noted in Section 3.1 is of a form that such an interaction cannot represent. The observed difference between the two is −110, −909 and +162 kN at 17, 24 and 30 storeys and therefore does not vary monotonically with height, whereas an interaction between bracing type and the storey number as a continuous variable can only produce a monotonic change, here −398, −278 and −175 kN. A separate bracing effect for each height group does reproduce the observed values, −112, −896 and +155 kN, but it raises the effective number of parameters to 12.9 for 45 observations and does not improve prediction. The additive model was therefore retained for the fitted relationship, and the height-dependent differences between bracing types are reported from the observations in Section 3.1.

The form of the model was also examined for nonlinearity beyond the terms listed in Table 12. Two of the three configuration variables are categorical and are represented exactly by the one-hot encoding, so the only variable for which a nonlinear form could be defined is the storey number, and it takes three values. Any function of three values has at most three degrees of freedom, which the quadratic term of Table 12 already spans, so a spline or a generalized additive model has nothing further to fit for this variable. The equivalent saturated form, a separate constant for each height group, is the factor coding reported in Section 2.3; on the leave-one-out predictions it gives a mean absolute error of 421 kN against 410 kN for the linear form, which is the more accurate on 24 of the 45 configurations (*p* = 0.03). The simplicity of the retained model is therefore a property of the design rather than a restriction imposed by the size of the dataset.

Taken together, Figure 4, Figure 5, Figure 6, Figure 7 and Figure 8 and Table 8, Table 9, Table 10, Table 11 and Table 12 show that the relationship between the three configuration variables and the best-observed feasible structural weight can be represented with useful accuracy within the investigated design domain. Random Forest provides the smallest mean absolute prediction errors, whereas Ridge provides an almost identical mean test *R*^2^, lower fold-to-fold variability, and a directly interpretable equation. The reference Ridge model is therefore most appropriately used as a preliminary screening tool for comparing candidate configurations within the parameter range represented by the optimization dataset. It does not replace code-compliant structural analysis or constrained optimization when a final member design is required.

## 4. Conclusions

This study examined whether the outcome of computationally intensive minimum-weight optimization of braced high-rise steel frames can be estimated directly from a small set of configuration variables. Forty-five combinations of storey number, bracing type, and brace-placement layout were analysed using HBA, AO, GWO, and PSO, giving 180 optimization runs. The lowest feasible weight obtained from the four searches for each configuration was retained as the best-observed feasible weight and used to develop the machine-learning models. The results demonstrate that the optimization outcome depends jointly on structural configuration and search algorithm; no single metaheuristic consistently produced the lightest feasible design.

PSO produced the lowest observed weight in 25 configurations and shared one additional minimum with GWO, while AO, GWO, and HBA produced the lowest values in ten, seven, and two configurations, respectively. This variation confirms the benefit of using more than one search strategy when generating optimization-based training data. A Friedman test over the 45 matched configurations rejects equal performance among the four algorithms (*p* = 3.8 × 10^−13^), and all six pairwise comparisons remain significant after Holm correction, with mean ranks of 1.68 for PSO, 2.10 for GWO, 2.53 for AO and 3.69 for HBA. Across the investigated height groups, the mean best-observed feasible weight increased from 2537 kN at 17 storeys to 4983 kN at 24 storeys and 7103 kN at 30 storeys. Bracing type produced a larger design effect than brace-placement layout. X-bracing yielded the highest mean weight at all three heights, whereas K-bracing gave the lowest mean weight at 17 and 24 storeys. At 30 storeys, however, the mean weights of V- and K-bracing differed by only about 2.4%, showing that the preferred bracing system cannot be selected independently of building height. The influence of bracing type reached approximately 30% of the mean weight at 24 storeys, whereas the effect of brace-placement layout remained within approximately 10–15%. The most favourable layout also changed with height, confirming that brace distribution should be examined together with brace type rather than prescribed in advance.

Among the six regression models, Ridge and Random Forest provided the strongest predictive performance but according to different error measures. Ridge achieved the highest mean five-fold cross-validated *R*^2^ of 0.914, while Random Forest produced the lowest mean test RMSE and MAE, at 511.7 and 343.1 kN, respectively. The difference in mean *R*^2^ between Ridge and Random Forest was only 0.001 and does not establish a statistically meaningful superiority of one model over the other. A paired Wilcoxon signed-rank test on the leave-one-out absolute errors gives *p* = 0.26. Ridge was retained as the reference model because it combined comparable predictive accuracy with lower fold-to-fold variability and a directly interpretable additive equation. The poorer test performance of Extra Trees, XGBoost, and KNN despite their near-perfect or perfect training fits also shows that additional model flexibility was not advantageous for this dataset of only 45 configurations.

The fitted Ridge model identified storey number as the dominant predictor, followed by bracing type, while brace-placement layout had a smaller average effect. This ordering is consistent with the optimization results, although the storey-number coefficient should not be interpreted as a height effect: the three groups differ in loading, in storey height and in plan geometry, and the grouping accounts for 90.8% of the total variation in the target. The additive form of Ridge also represents average effects over the investigated configurations and cannot reproduce all height-dependent changes in the relative performance of V-, X-, and K-bracing. These qualifications are important when the fitted equation is used for preliminary comparisons.

Bootstrap refitting of the Ridge model provided an additional measure of model sensitivity. Seven of the nine hold-out cases had coefficients of variation below 6%, while the two lowest-weight cases showed values of approximately 10–17%; the mean CV over the hold-out set was approximately 7.5%. Evaluated over all 45 configurations, the sensitivity of the predictions to the sample of configurations corresponds to a mean standard deviation of 224 kN, and the stochastic optimization that defines the target to 248 kN; both are smaller than the leave-one-out mean absolute error of 410 kN. The structural model, the loading, and the material properties are common to the configurations of a height group, so they do not affect comparisons within a group, where the model orders 69.5% of the pairs correctly, rising to 83% for differences above 1000 kN. Differences smaller than the prediction error, such as the approximately 2.4% difference between V- and K-bracing at 30 storeys, are resolved by carrying out the optimization for the shortlisted configurations.

The principal outcome of the study is that a simple regression model can reproduce the best-observed feasible weights of the investigated braced-frame configurations with sufficient accuracy to support preliminary screening without repeating the full optimization procedure for every alternative. The model is intended to narrow the set of configurations requiring detailed analysis rather than replace code-based structural analysis or constrained member optimization. Its present applicability is limited to the investigated range of 17–30 storeys; V-, X-, and K-bracing; the five brace-placement layouts; the adopted member grouping; and the adopted loading and modelling assumptions.

Several directions follow for further work: a consistent wind-loading strategy at all three heights; frames designed under the seismic provisions of AISC 341; repeated runs of each algorithm with several random seeds, which would also allow a distributional target to be predicted; alternative penalty exponents and member groupings; and storey numbers and brace-placement layouts outside the present factorial arrangement. The framework can also be extended to fire exposure, which would require the temperature dependence of the material properties, the stiffness and the distribution of forces to be represented and could change both the governing constraints and the bracing arrangement that minimizes weight; thermal finite-element analysis of members under standardized fire, reported for reinforced concrete beams in [78], indicates the kind of input such an extension would require.

## Figures and Tables

**Table 1:** Representative studies related to optimization and ML-assisted design of steel framing systems.

Study	Structural Problem	Approach	Main Finding or Contribution	Relation to the Present Study
Üstüner and Doğan [12]	Outrigger-braced high-rise steel frames	Metaheuristic weight optimization	Examined the effect of stiffening configuration on minimum-weight design while considering soil-structure interaction	Each structural configuration requires separate optimization
Zhu et al. [16]	Centrally braced steel frames	Brace-arrangement optimization	Demonstrated that brace location affects structural response and optimization outcome	No direct ML prediction of the optimized structural weight
Üstüner and Doğan [21]	Real-scale high-rise steel buildings	Minimum-weight optimization of alternative outrigger arrangements	Showed that stiffening arrangement affects the minimum-weight solution	Alternative arrangements remain dependent on repeated optimization
Bekdaş et al. [26]	Structural design problems	ML trained using optimization results	Predicted optimum design variables from previously generated optimization data	Applied to comparatively simple structural systems
Shan et al. [27]	Steel frames	Integrated optimization and ML	Used ML within an intelligent structural-design procedure	Optimization remains part of the solution process
Truong et al. [28]	Nonlinear steel structures	XGBoost surrogate and multi-objective optimization	Reduced expensive constraint evaluations during optimization	Predicts constraint response rather than the optimized structural weight
Shehzad et al. [29]	High-rise outrigger systems	ML and genetic optimization	Used learned response relationships to identify effective structural configurations	Emphasis placed mainly on seismic performance
Vu et al. [30]	Nonlinear steel trusses	Regression and classification surrogates	Reduced repeated nonlinear analyses during sizing optimization	Optimization loop retained
Wang et al. [31]	Steel–concrete composite structures	Review of ML applications	Reported increasing use of regression and ensemble learning for structural response and design prediction	Provides broader evidence for ML-assisted structural engineering
Present study	Braced high-rise steel frames, 17–30 storeys	Regression models trained on completed metaheuristic optimization results	Best-observed feasible weight predicted directly from storey number, bracing type, and brace-placement layout	Predicts the optimization outcome itself; no optimization run is required to screen a configuration within the investigated domain

**Table 2:** Search parameters adopted for the metaheuristic algorithms.

Algorithm	Parameter	Adopted Value
HBA	Density-factor coefficient, *C*	2
HBA	Digging ability coefficient, *β*	6
AO	Exploitation coefficient, *α*	0.1
AO	Exploitation coefficient, *δ*	0.1
AO	Spiral coefficient, *ω*	0.005
AO	Spiral coefficient, *U*	0.00565
GWO	Convergence parameter, *a*	Linear decrease from 2 to 0
PSO	Cognitive and social coefficients, *c*_1_, *c*_2_	2, 2
PSO	Inertia weight, *ω*	1
All algorithms	Population size	30
All algorithms	Maximum number of iterations	50,000

**Table 3:** Structural weights obtained for the 17-storey configurations using the four metaheuristic algorithms.

Brace Type	Layout	HBA (kN)	GWO (kN)	AO (kN)	PSO (kN)
V	All	3357.571	3114.339	2297.501	3017.977
V	1-1	3319.580	2928.722	2577.577	3173.197
V	2-2	4764.152	2569.333	2326.277	2640.781
V	2-1	5643.506	3226.632	3153.094	2571.271
V	3-1	5848.862	2946.576	5186.312	2742.329
X	All	3782.230	2766.319	2596.499	2793.761
X	1-1	3034.756	2969.938	2816.752	2657.886
X	2-2	5349.741	3166.224	3209.359	2987.216
X	2-1	3681.697	2935.466	2547.580	2759.269
X	3-1	5375.543	2910.364	3336.656	2790.571
K	All	2295.108	2801.928	2545.957	2546.722
K	1-1	3249.896	2817.828	2515.170	2743.810
K	2-2	2987.675	2496.030	2264.085	2594.988
K	2-1	2467.556	2712.740	2534.527	3225.806
K	3-1	5902.141	2431.744	2424.335	2810.787

**Table 4:** Structural weights obtained for the 24-storey configurations using the four metaheuristic algorithms.

Brace Type	Layout	HBA (kN)	GWO (kN)	AO (kN)	PSO (kN)
V	All	12,020.936	6039.048	6140.031	6039.048
V	1-1	10,395.157	4137.593	6668.890	4461.473
V	2-2	8553.115	5435.178	6782.362	5126.680
V	2-1	8090.735	5881.365	5883.519	4382.259
V	3-1	8202.388	6072.234	6274.877	5735.551
X	All	16,255.154	6698.710	8898.256	5374.109
X	1-1	14,517.037	6287.398	6402.837	7341.277
X	2-2	8178.847	4877.961	7163.074	5722.040
X	2-1	9948.695	8127.377	16,913.223	6341.168
X	3-1	13,354.509	6193.730	13,346.897	5571.559
K	All	7287.375	6324.413	7181.967	4807.658
K	1-1	10,681.434	5630.826	6132.146	3502.945
K	2-2	8070.470	5515.537	6076.280	4280.872
K	2-1	8027.370	5502.332	6526.091	3718.091
K	3-1	7086.723	5579.302	7626.772	4565.761

**Table 5:** Structural weights obtained for the 30-storey configurations using the four metaheuristic algorithms.

Brace Type	Layout	HBA (kN)	GWO (kN)	AO (kN)	PSO (kN)
V	All	8378.162	6803.341	6784.618	7176.580
V	1-1	9431.103	6932.129	12,051.111	7395.945
V	2-2	7771.129	6718.141	7274.643	6366.247
V	2-1	7759.826	6853.980	7980.554	6857.879
V	3-1	7077.305	6625.759	7661.469	6881.831
X	All	8584.066	8201.376	7824.041	8119.084
X	1-1	12,069.524	7970.160	8148.861	9012.735
X	2-2	8706.106	7321.451	7812.796	7303.126
X	2-1	9300.522	7852.401	8226.989	7817.552
X	3-1	9016.332	8409.478	8371.776	7692.549
K	All	11,056.085	6955.744	7161.498	6799.880
K	1-1	7829.615	8193.162	8050.257	7470.913
K	2-2	8264.833	6800.953	7019.328	6470.841
K	2-1	8134.345	7172.757	7338.144	6833.427
K	3-1	7739.252	7041.257	7922.767	6799.181

**Table 6:** Summary of the weights obtained by each metaheuristic algorithm over the 45 configurations.

Algorithm	Mean Weight (kN)	Standard Deviation (kN)	Lowest Weight (kN)	Highest Weight (kN)
PSO	5067.2	1975.6	2546.7	9012.7
GWO	5354.4	1984.9	2431.7	8409.5
AO	6221.7	3107.0	2264.1	16,913.2
HBA	7618.8	3214.1	2295.1	16,255.2

**Table 7:** Hyperparameters used for the regression models.

Model	Parameter	Adopted Value
Ridge	Regularization strength, α	0.1
	Solver	auto
	Tolerance	1 × 10^−4^
Random Forest	Number of estimators	500
	Random state	0
	Maximum depth	None
	Minimum samples per leaf	1
Extra Trees	Number of estimators	500
	Random state	0
	Maximum depth	None
	Minimum samples per leaf	1
AdaBoost	Number of estimators	200
	Random state	0
	Learning rate	1.0
	Base estimator	Decision tree, maximum depth = 3
XGBoost	Number of estimators	500
	Learning rate	0.05
	Maximum tree depth	3
	Subsample ratio	0.9
	Random state	0
	Minimum child weight	1
	L_2_ regularization, λ	1
KNN	Number of neighbours, *k*	3
	Weighting scheme	Distance
	Distance metric	Minkowski, *p* = 2
	Algorithm	auto

**Table 8:** Predictive performance of the regression models obtained by five-fold cross-validation.

		Train Data				Test Data			
Model	Fold	*R* ^2^	RMSE	MSE	MAE	*R* ^2^	RMSE	MSE	MAE
Ridge	1	0.943	463.940	215,240.470	366.055	0.963	388.550	150,971.172	280.107
	2	0.957	422.616	178,603.892	333.287	0.856	573.860	329,315.736	428.339
	3	0.947	443.411	196,612.915	335.678	0.941	486.016	236,211.953	401.717
	4	0.959	399.806	159,845.226	309.735	0.868	666.178	443,793.376	504.696
	5	0.944	446.610	199,460.169	344.577	0.944	500.223	250,222.809	375.036
	Mean	0.950	435.276	189,952.535	337.866	0.914	522.966	282,103.009	397.979
Random Forest	1	0.986	230.474	53,118.247	155.667	0.980	284.929	81,184.327	202.348
	2	0.993	164.966	27,213.621	112.185	0.832	619.661	383,980.113	502.295
	3	0.988	211.384	44,683.316	147.605	0.935	509.731	259,825.478	250.924
	4	0.990	200.832	40,333.518	131.224	0.873	651.835	424,888.572	422.571
	5	0.989	195.498	38,219.596	147.013	0.946	492.254	242,313.956	337.435
	Mean	0.989	200.631	40,713.660	138.739	0.913	511.682	278,438.489	343.114
AdaBoost	1	0.961	383.023	146,706.892	253.416	0.919	571.844	327,005.448	417.375
	2	0.987	234.082	54,794.205	167.664	0.742	766.924	588,172.423	593.465
	3	0.975	306.704	94,067.190	211.299	0.935	512.898	263,064.541	333.404
	4	0.976	304.711	92,848.956	218.824	0.913	540.694	292,349.853	369.341
	5	0.976	295.253	87,174.172	219.864	0.943	505.192	255,218.939	395.983
	Mean	0.975	304.755	95,118.283	214.214	0.890	579.510	345,162.241	421.913
Extra Trees	1	1.000	0.000	0.000	0.000	0.970	346.334	119,946.985	238.996
	2	1.000	0.000	0.000	0.000	0.706	819.366	671,360.268	676.513
	3	1.000	0.000	0.000	0.000	0.909	606.153	367,421.889	323.208
	4	1.000	0.000	0.000	0.000	0.809	800.144	640,230.485	458.614
	5	1.000	0.000	0.000	0.000	0.872	755.770	571,187.978	430.894
	Mean	1.000	0.000	0.000	0.000	0.853	665.553	474,029.521	425.645
XGBoost	1	0.997	110.224	12,149.342	94.669	0.938	500.636	250,636.321	430.337
	2	0.999	54.799	3002.879	43.963	0.642	903.998	817,212.928	802.778
	3	0.997	103.311	10,673.071	82.032	0.897	643.928	414,643.032	489.335
	4	0.999	44.669	1995.303	32.393	0.781	857.424	735,175.876	670.694
	5	0.998	75.129	5644.310	60.082	0.900	669.548	448,293.882	475.483
	Mean	0.998	77.626	6692.981	62.628	0.832	715.107	533,192.408	573.725
KNN	1	1.000	0.000	0.000	0.000	0.841	800.572	640,916.304	706.823
	2	1.000	0.000	0.000	0.000	0.555	1007.991	1,016,046.435	874.795
	3	1.000	0.000	0.000	0.000	0.785	929.528	864,021.766	875.581
	4	1.000	0.000	0.000	0.000	0.780	859.726	739,128.861	732.012
	5	1.000	0.000	0.000	0.000	0.724	1111.034	1,234,396.631	980.167
	Mean	1.000	0.000	0.000	0.000	0.737	941.770	898,901.999	833.876

**Table 9:** Test *R*^2^ of the regression models obtained from 1000 repetitions of five-fold cross-validation.

Model	Mean Test *R*^2^	Standard Deviation	2.5th Percentile	97.5th Percentile
Ridge	0.904	0.022	0.859	0.927
Random Forest	0.899	0.033	0.826	0.936
AdaBoost	0.896	0.038	0.819	0.939
Extra Trees	0.824	0.050	0.725	0.890
XGBoost	0.821	0.049	0.712	0.878
KNN	0.752	0.058	0.618	0.832

**Table 10:** Effect of removing one algorithm from the definition of the response variable, obtained from 200 repetitions of five-fold cross-validation.

Algorithm Removed	Configurations with Unchanged Target	Mean Increase in Target (%)	Spearman Correlation with Full Target	Mean Test *R*^2^
HBA	43 of 45	0.3	0.998	0.902
GWO	38 of 45	0.9	0.996	0.911
AO	35 of 45	2.1	0.983	0.895
PSO	20 of 45	8.5	0.965	0.834

**Table 11:** Prediction error for a factor level withheld from the training set.

Held-Out Level	Cases	MAE (kN)	MAE (% of Group Mean)	Mean Signed Error (kN)
17 storeys	15	379	14.9	−15
24 storeys	15	631	12.7	+12
30 storeys	15	311	4.4	−34
K-bracing	15	608	13.6	+590
V-bracing	15	453	9.5	+162
X-bracing	15	755	14.0	−752
Layout 1-1	9	539	11.0	−25
Layout 2-1	9	414	8.6	+47
Layout 2-2	9	332	7.1	+259
Layout 3-1	9	272	5.4	−150
Layout All	9	317	6.4	−132

**Table 12:** Predictive performance of alternative model forms.

Model Form	Effective Parameters	Mean Test *R*^2^	Leave-One-Out MAE (kN)	Paired *p* Against the Reference
Additive (reference)	7.94	0.904	410	-
With height × bracing interaction	9.93	0.908	379	0.11
With height × layout interaction	11.90	0.891	433	0.28
With both interactions	13.88	0.896	391	0.87
With quadratic height term	8.93	0.893	424	<0.001
Separate bracing effect per height group	12.86	0.901	398	0.79

## Data Availability

The original contributions presented in this study are included in the article. Further inquiries can be directed to the corresponding author.
